# A structural homologue of the plant receptor D14 mediates responses to strigolactones in the fungal phytopathogen *Cryphonectria parasitica*

**DOI:** 10.1111/nph.18013

**Published:** 2022-02-26

**Authors:** Valentina Fiorilli, Marco Forgia, Alexandre de Saint Germain, Giulia D’Arrigo, David Cornu, Philippe Le Bris, Salim Al‐Babili, Francesca Cardinale, Cristina Prandi, Francesca Spyrakis, François‐Didier Boyer, Massimo Turina, Luisa Lanfranco

**Affiliations:** ^1^ Dipartimento di Scienze della Vita e Biologia dei Sistemi Università di Torino Viale P.A. Mattioli 25 Torino 10125 Italy; ^2^ Istituto per la Protezione Sostenibile delle Piante – CNR Strada delle Cacce 73 10135 Torino Italy; ^3^ INRAE, AgroParisTech Institut Jean‐Pierre Bourgin (IJPB) Université Paris‐Saclay 78000 Versailles France; ^4^ Dipartimento di Scienza e Tecnologia del Farmaco Università di Torino via P. Giuria 11 10125 Torino Italy; ^5^ CEA, CNRS Institute for Integrative Biology of the Cell (I2BC) Université Paris‐Saclay 1 Avenue de la Terrasse 91198 Gif‐sur‐Yvette France; ^6^ Division of Biological and Environmental Science and Engineering King Abdullah University of Science and Technology Thuwal 23955‐6900 Saudi Arabia; ^7^ Dipartimento di Scienze Agrarie, Forestali e Alimentari Università di Torino Largo Braccini 2 10095 Grugliasco Italy; ^8^ Dipartimento di Chimica Università di Torino via P. Giuria 7 10125 Torino Italy; ^9^ CNRS Institut de Chimie des Substances Naturelles UPR 2301 Université Paris‐Saclay 1 Avenue de la Terrasse 91198 Gif‐sur‐Yvette France

**Keywords:** α/β‐hydrolase, apocarotenoids, *Cryphonectria parasitica*, DWARF14 (D14), fungus, perception, strigolactones

## Abstract

- Strigolactones (SLs) are plant hormones and important signalling molecules required to promote arbuscular mycorrhizal (AM) symbiosis. While in plants an α/β‐hydrolase, DWARF14 (D14), was shown to act as a receptor that binds and cleaves SLs, the fungal receptor for SLs is unknown.
- Since AM fungi are currently not genetically tractable, in this study, we used the fungal pathogen *Cryphonectria parasitica*, for which gene deletion protocols exist, as a model, as we have previously shown that it responds to SLs. By means of computational, biochemical and genetic analyses, we identified a D14 structural homologue, CpD14.
- Molecular homology modelling and docking support the prediction that CpD14 interacts with and hydrolyses SLs. The recombinant CpD14 protein shows α/β hydrolytic activity *in vitro* against the SLs synthetic analogue GR24; its enzymatic activity requires an intact Ser/His/Asp catalytic triad. CpD14 expression in the *d14‐1* loss‐of‐function *Arabidopsis thaliana* line did not rescue the plant mutant phenotype. However, gene inactivation by knockout homologous recombination reduced fungal sensitivity to SLs.
- These results indicate that CpD14 is involved in SLs responses in *C. parasitica* and strengthen the role of SLs as multifunctional molecules acting in plant–microbe interactions.

## Introduction

*Cryphonectria parasitica* is a bark pathogen, which causes perennial necrotic lesions (cankers) on the above‐ground part of susceptible host trees. This fungus is still a major threat to American and European chestnut trees causing the blight disease, which leads to yield losses of fruit and wood (Rigling & Prospero, 2018).

*Cryphonectria parasitica* is a model fungus for studying how mycovirus causes the hypovirulent phenotype, and a wealth of different approaches has only partially elucidated how the severity of disease can be tempered by the presence of *Cryphonectria hypovirus 1* (CHV1) (Eusebio‐Cope *et al*., 2015). Notwithstanding the detailed account of virus‐caused perturbation characterized using different approaches (Choi & Nuss, 1992; Allen *et al*., 2003; Dawe *et al*., 2009; Wang *et al*., 2014), not much is known about the possible molecular signalling occurring between the plant and the fungus, resulting in pathogenicity (Lovat & Donnelly, 2019): no specific gene products, structural component or metabolite has been identified as playing a role in the mutual recognition between plant and fungus, and novel molecules could be hypothesized among essential or accessory players.

A few years ago, we demonstrated that *C. parasitica* is sensitive to (±)‐GR24 (GR24 hereafter) (Belmondo *et al*., 2017), a synthetic analog of strigolactones, confirming what was also observed in several other plant pathogenic fungi (Dor *et al*., 2011).

Strigolactones (SLs) are a class of carotenoid‐derived phytohormones (Al‐Babili & Bouwmeester, 2015), characterized by a methylbutenolide moiety (D‐ring) linked to a variable tricyclic lactone (ABC‐ring system). SLs, which play a role in the regulation of several aspects of plant growth and development and responses to biotic and abiotic stress (Gomez‐Roldan *et al*., 2008; Umehara *et al*., 2008; Ha *et al*., 2014; Lopez‐Obando *et al*., 2015; Waters *et al*., 2017; Koltai & Prandi, 2019), were first described as plant metabolites able to stimulate seed germination in parasitic plants (Cook *et al*., 1966). In addition, when released by roots into the soil, SLs also enhance the growth and metabolism of a group of beneficial symbiotic fungi (Akiyama *et al*., 2005; Besserer *et al*., 2006, 2008; Genre *et al*., 2013; Salvioli *et al*., 2016; Tsuzuki *et al*., 2016; Kamel *et al*., 2017; Volpe *et al*., 2020), favouring the establishment of arbuscular mycorrhizal (AM) symbiosis, a very ancient and widespread mutualistic association with plant roots (MacLean *et al*., 2017; Lanfranco *et al*., 2018a).

Strigolactones seem therefore multifunctional molecules active on plants and microorganisms (Lopez‐Raez *et al*., 2017; Schlemper *et al*., 2017; Lanfranco *et al*., 2018b; Carvalhais *et al*., 2019; Rochange *et al*., 2019).

To our knowledge, the mechanism of SLs perception in fungi is not known. On the contrary, SLs perception has been elucidated in plants (Hamiaux *et al*., 2012; Waters *et al*., 2017): the rice DWARF14 (D14) protein was described as a noncanonical receptor having the dual function of enzyme and receptor (de Saint Germain *et al*., 2016; Yao *et al*., 2016). D14 is an α/β‐hydrolase that can bind and cleave SLs, by performing a nucleophilic attack on the D‐ring. However, the hydrolytic event is currently debated as an essential process that promotes activation of D14 (Shabek *et al*., 2018; Seto *et al*., 2019; Bürger & Chory, 2020). Upon SL binding, D14 undergoes a conformational change to recruit downstream signalling components, such as the D3 protein, for triggering SL responses (Yao *et al*., 2018). A similar irreversible perception mechanism was proven for D14 homologues from other plant species including Arabidopsis (AtD14 Chevalier *et al*., 2014), pea (RAMOSUS3 (RMS3) de Saint Germain, 2016), petunia (DECREASED APICAL DOMINANCES (DAD2) Hamiaux *et al*., 2012) and the root parasitic weed *Striga hermonthica* (ShHYPOSENSITIVE TO LIGHT7 (ShHTL7) Toh *et al*., 2015; Yao *et al*., 2017). A clear D14 homologue was not found in the genome of AM fungi (Tisserant *et al*., 2013; Lin *et al*., 2014), the most studied fungal system for SLs responses.

In this context, we decided to exploit the SLs‐sensitive fungus *C*. *parasitica* for which, in contrast to AM fungi, stable genetic transformation protocols are available.

In this work, we tested the hypothesis that fungi possess D14 homolog proteins that could bind and hydrolyse SLs and that could eventually complement the mutation of the corresponding plant homologue. We identified a candidate D14 structural homologue in *C. parasitica* and called it CpD14; by means of molecular modelling analyses and biochemical characterization of the recombinant protein, we demonstrated CpD14 binding and enzymatic activity on natural SLs and their synthetic analogues. The expression of *CpD14* in the *d14* loss‐of‐function *Arabidopsis thaliana* line did not rescue the plant mutant phenotype. However, gene inactivation by knockout homologous recombination reduced *C. parasitica* sensitivity to SLs, indicating that the gene is indeed involved in SLs responses in this fungus.

## Materials and Methods

### Fungal strains

*Cryphonectria parasitica* (Murrill) Barr. Δ*cpku80*, in this work considered a wild‐type (WT) strain, was chosen for its ability to generate double homologous recombination and enhance site‐directed mutagenesis efficiency (Lan *et al*., 2008).

### Generation of *C. parasitica CpD14*‐mutant strains

*CpD14* knockout strains (Δ*cpd14_48*, *Δcpd14_137* and Δ*cpd14_141*) were generated by site‐directed double homologous recombination with a construct carrying a hygromycin B resistance cassette. The *CpD14* gene, including *c*. 1 kb upstream and downstream sequences, was amplified using KOFor and KORev primers (Supporting Information Table S1). The 3477‐bp‐long amplicon was cloned in the pCR Zero blunt vector. The recombinant plasmid was digested with *Afe*I to delete a 293‐bp portion in the gene sequence. The hygromycin B resistance cassette was amplified through PCR from the pCB1004 vector (Carroll *et al*., 1994) using the Gibson assembly primer couple GibFor and GibRev (Table S1), and the PCR product was cloned in the digested plasmid through the Takara Infusion kit. The knockout plasmid was linearized by restriction and transformed into protoplasts of the *C. parasitica* Δ*cpku80* strain. Hygromycin‐resistant colonies were screened through PCR using primers amplifying full‐length *CpD14*. Transformed colonies where double homologous recombination had occurred gave a 2000‐bp band instead of the normal PCR product of *c*. 1000 bp. Selected mutants were grown on potato dextrose agar (PDA) media; conidia were harvested from 1‐month‐old cultures and plated to obtain single conidia colonies. A further PCR screening was carried out on single conidia colonies from each independent mutant using primers as forward the KOFor primer and as reverse the hygromycin resistance cassette Hyg100For (Table S1), leading to a PCR product of *c*. 1500 bp.

### Fungal growth assays

*Cryphonectria parasitica* strains were precultivated for 3 d at 20°C on solid PDA medium. All standard cultivations were done under diurnal light conditions. Afterwards, one mycelial plug was transferred on solid B5 medium (Duchefa, 3.17 g l^−1^) supplemented with 2% glucose as described in Fiorilli *et al*. (2021). The screening was carried out in 3.5‐cm microtiter wells (7 ml medium per well). Stock solutions of (+)‐2′‐*epi*‐GR24; (−)‐GR24; (+)‐GR24; (−)‐2′‐*epi*‐GR24 (MW 298.29) and of tolfenamic acid (an inhibitor of plant SLs receptors; Hamiaux *et al*., 2018; MW 261.71) were prepared dissolving the powder in the appropriate solvent (acetone for GR24 stereoisomers and dimethyl sulfoxide (DMSO) for tolfenamic acid) to obtain a 10 mM solution. The WT strain was analysed in triplicate on (+)‐2′‐*epi*‐GR24; (−)‐GR24; (+)‐GR24; (−)‐2′‐*epi*‐GR24 (from 10^−4^ to 10^−5^ M) and acetone control in parallel. WT and Δ*cpd14* mutant strains were analysed in triplicate on (+)‐GR24 (10^−4^ M), on tolfenamic acid (10^−6^ M), on both (+)‐GR24 (10^−4^ M) and tolfenamic acid (10^−6^ M) and in parallel on acetone, DMSO, acetone + DMSO as corresponding control samples. Microtiter wells were kept in a dark room at 20°C, and at 24, 48, 72 and 96 h, the colony diameter of the hyphal radial growth was measured. No effect of the solvents was observed (data not shown). Pictures were taken at the edge of the colony, 96 h post‐inoculation using a light microscope Primo Star Zeiss (Carl Zeiss MicroImaging, Göttingen, Germany) with a Leica DFC425 digital camera attached (Leica Microsystems, Wetzlar, Germany) to highlight possible alterations in hyphal growth and morphology.

### Molecular homology modelling and docking

The CpD14 sequence was used as a query to perform a template search in the Protein Data Bank (PDB) database with the PSI‐Blast algorithm. The DAD2 X‐ray structure (PDB code: 4dnp) was identified as the best possible template, according to the sequence identity and coverage, and used to build the CpD14 three‐dimensional (3D) model with the SWISS‐MODEL server (Waterhouse *et al*., 2018). The quality assessment of the predicted model was validated by building the Ramachandran plot using the RAMPAGE server (http://mordred.bioc.cam.ac.uk/~rapper/rampage.php).

Docking simulations with natural and synthetic analogues of SLs were performed using the GOLD suite v.5.5, as done previously (Lombardi *et al*., 2017; Sanchez *et al*., 2018). The pocket of interest was defined to contain all the residues within 10 Å from a reference atom (CD2, Leu188). Due to some protruding residues in the ligand‐binding pocket, and to ensure a correct positioning of the compounds into the pocket, side‐chain flexibility was allowed to the bulkiest residues (Tyr164, Glu227 and Met253). Moreover, to reproduce the catalytic environment, Ser104 and His181 were simulated, respectively, in the deprotonated and protonated state. (+)‐GR24, (−)‐2′‐*epi*‐GR24 and (+)‐strigol were submitted to 15 genetic algorithm runs, and the CHEMPLP score function was used to select the best pose.

Molecular dynamics (MD) simulation of the CpD14 model was performed using the GROMACS v.4.6.1 (Pronk *et al*., 2013). Protein topology was generated with the Amber99SBILDN force field (Hornak, *et al*., 2006), and the protein was solvated using the TIP3P water molecules (Jorgensen *et al*., 1983) in a 12 Å cubic box. The box and the MD set‐up were built using the BiKi Life Sciences suite (Spyrakis *et al*., 2015; Decherchi *et al*., 2018). The system was subjected to 5000 steps of energy minimization with the steepest descent algorithm. Then, four equilibration steps were carried out: 500 ps in the NVT ensemble at 100, 200 and 300 K, and 1 ns in the NPT ensemble with an integration step of 2 fs. Finally, the system was simulated for 200 ns in the NVT ensemble.

### Cloning, expression and purification of recombinant proteins

The *CpD14* full‐length cDNA was amplified from the RNA of *C. parasitica* grown in liquid cultures using a forward primer (CpD14‐F) containing a *Bgl*II site and a reverse primer (CpD14‐R) containing an *Kpn*I site (Table S1); the sequence was cloned into the pHUE vector. To obtain the mutated version of the protein, the vector pHUE containing the complete coding sequence of *CpD14* was amplified through PCR using a forward primer carrying two mutated nucleotides. The forward primer (CpD14For) was used with the reverse primer 309Rev (Table S1) to amplify a linearized full‐length plasmid carrying the mutation in positions 309 and 310 to change the codon usage from Ser to Ala. The obtained linearized plasmid was phosphorylated and ligated with a T4 ligase (Thermo Fisher Scientific, Waltham, MA, USA). The CpD14^S104A^‐specific mutation was confirmed through sequencing.

For CpD14 protein expression, the full‐length coding sequences from *C. parasitica* were amplified by PCR using the recombinant plasmid pHUE‐*CpD14* as template and specific primers (CpD14_attb1_HRV3C and CpD14_attb2 (Table S1) containing a protease cleavage site for tag removal, and subsequently cloned into the pGEXT‐4T‐3 expression vector).

### Preparation of GR24 isomers and GC probes

(+)‐GR24, (−)‐GR24, (+)‐2′‐*epi*‐GR24 and (‐)‐2′‐*epi*‐GR24 were separated from (±)‐2′‐*epi*‐GR24 and (±)‐GR24 by chiral supercritical fluid chromatography as described in de Saint Germain *et al*. (2016, 2019). Probes (GC486, GC240 and GC242) were prepared also according to de Saint Germain *et al*. (2016).

### Enzymatic degradation of GR24 isomers by purified proteins

The ligand (10 µM) was incubated with and without purified proteins (5 µM) for 150 min at 25°C in 0.1 ml phosphate buffer saline (PBS – 100 mM phosphate, pH 6.8, 150 mM NaCl) in the presence of (±)‐1‐indanol (100 µM) as internal standard. The solutions were acidified to pH 1 by the addition of trifluoroacetic acid (2 µl) to quench the reaction and centrifugated (12 min, 10 000 ***g***). The samples were subjected to RP‐UPLC‐MS analyses using a UPLC system equipped with a PDA and a triple quadrupole mass spectrometer detector (Acquity UPLC‐TQD; Waters, Milford, MA, USA). RP‐UPLC (HSS C_18_ column, 1.8 μm, 2.1 mm × 50 mm) with 0.1% formic acid in CH_3_CN and 0.1% formic acid in water (aq. FA, 0.1%, v/v, pH 2.8) as eluents (10% CH_3_CN, followed by linear gradient from 10% to 100% of CH_3_CN (4 min)) at a flow rate of 0.6 ml min^−1^. The detection was performed by PDA and using the TQD mass spectrometer operated in electrospray ionization positive mode at 3.2 kV capillary voltage. The cone voltage and collision energy were optimized to maximize the signal and were, respectively, 20 V for cone voltage and 12 eV for collision energy, and the collision gas was argon at a pressure maintained near of 4.5 × 10^−3^ mBar.

### Enzymatic assay with profluorescent probes

These assays were performed as described in de Saint Germain *et al*. (2016), using a TriStar LB 941 Multimode Microplate Reader (Berthold Technologies, Bad Wildbad, Germany).

### Temperature melts proteins *nanoDSF*

Proteins were diluted in PBS to *c*. 10 µM concentration. Ligand was tested at the concentration of 200 µM. The intrinsic fluorescence signal was measured as a function of increasing temperature in Prometheus NT.48 fluorimeter (Nanotemper™), with 55% excitation light intensity and 1°C min^−1^ temperature ramp. Analyses were performed on capillaries filled with 10 µl of respective samples. Intrinsic fluorescence signal expressed by the 350 nm : 330 nm emission ratio, which increases as the proteins unfold, is plotted as a function of temperature. The plots are one of the three independent data collections that were performed for each protein.

### Intrinsic tryptophan fluorescence assays and determination of the dissociation constant *K_D_*

These assays have been performed as described in de Saint Germain *et al*. (2016), using Spark^®^ Multimode Microplate Reader from Tecan.

### Direct ESI‐MS in denaturant conditions

Mass spectrometry measurements were performed with an electrospray Q‐TOF mass spectrometer (Waters) equipped with the Nanomate device (Advion Inc., Ithaca, NY, USA). The HD_A_384 chip (5 µm I.D. nozzle chip, flow rate range 100–500 nl min^−1^) was calibrated before use. For ESI‐MS measurements, the Q‐TOF instrument was operated in RF quadrupole mode with the TOF data being collected between *m/z* 400 and 2990. Collision energy was set to 10 eV, and argon was used as collision gas. Mass spectra acquisition was performed after denaturation of CpD14 ± ligand in 50% acetonitrile and 1% formic acid using Mass Lynx 4.1 (Waters) and Peakview 2.2 (Sciex). Deconvolution of multiply‐charged ions was performed by applying the MaxEnt algorithm (Sciex). The estimated mass accuracy is ± 2 Da. External calibration was performed with NaI clusters (2 µg µl^−1^, isopropanol : H_2_O, 50 : 50; Waters) in the acquisition *m/z* mass range.

### Localization of the fixation site of ligands on CpD14

RMS3‐ligand, CpD14‐ligand and CpD14^S104A^‐ligand mixtures were incubated for 10 min before being submitted overnight to Glu‐C proteolysis. Glu‐C‐generated peptide mixtures were analysed by nanoLC‐MS/MS with the Triple‐TOF 4600 mass spectrometer (AB Sciex) coupled to the nanoRSLC ultra‐performance liquid chromatography (UPLC) system (Thermo Scientific, Waltham, MA, USA) equipped with a trap column (Acclaim PepMap 100 C_18_, 75 µm i.d. × 2 cm, 3 µm) and an analytical column (Acclaim PepMap RSLC C_18_, 75 µm i.d. × 25 cm, 2 µm, 100 Å). Peptides were loaded at 5 µl min^−1^ with 0.05% TFA in 5% acetonitrile, and separation of peptides was performed at a flow rate of 300 nl min^−1^ with a 5–35% solvent B gradient in 40 min. Solvent A was 0.1% formic acid in water, and solvent B was 0.1% formic acid in 100% acetonitrile. NanoLC‐MS/MS experiments were conducted in a data‐dependent acquisition method by selecting the 20 most intense precursors for CID fragmentation with Q1 quadrupole set at low resolution for better sensitivity. Raw data were processed with MS Data Converter tool (AB Sciex), and protein identification was performed using MASCOT (Matrix Science, London, UK) against the CpD14 sequence with oxidation of methionine and ligand–histidine adduct as variable modifications. Peptide and fragment tolerance were, respectively, set at 20 ppm and 0.05 Da. Only peptides with mascot ions score above identity threshold (25) calculated at 1% FDR.

### Generation of *Arabidopsis thaliana* transgenic lines

The expression vectors for transgenic *Arabidopsis* were constructed by MultiSite Gateway Three‐Fragment Vector Construction kit (Invitrogen) as described in de Saint Germain *et al*. (2016). The *CpD14* CDS was tagged with 6xHA epitope tag at the C‐terminus and under control of *AtD14* native promoter (0.8 kb). *CpD14* CDS was PCR‐amplified from pGEX‐4T‐3‐CpD14 plasmid and recombined into *pDONR221* (Invitrogen). The suitable combination of *AtD14* promoters, *CpD14* CDS and 6xHA was cloned into the *pH7m34GW* final destination vectors by using three fragment recombination systems (Karimi *et al*., 2007) and named pD14::CpD14‐6xHA. Transformation of *Arabidopsi*s *Atd14‐1* mutant (kindly provided by M. Waters, University of Western Australia, Australia) was performed according to the conventional dipping method (Clough & Bent, 1998), with *Agrobacterium* strain GV3101. Phenotypic analysis and protein extraction were performed on the T3 homozygous lines.

### Plant phenotypic assays

Plants were grown in a glasshouse. Experiments were carried out in summer, under long photoperiods (15–16 h d^−1^); daily temperatures fluctuated between 18°C and 25°C. Peak levels of photosynthetically active radiation (PAR) were between 700 and 1000 μmol m^−2^ s^−1^. Plants were watered twice a week with tap water. The number of rosette leaves was counted just after bolting of the main shoot, and the number of rosette branches longer than 5 mm was counted when the plants were 40 d old.

### Expression profiles of *CpD14* and genes putatively involved in carotenoid biosynthesis and cleavage

To evaluate *CpD14* expression, WT and Δ*CpD14* mutant strains were grown in flasks containing B5 liquid media and kept in the dark on an orbital shaker for 96 h. After 96 h, liquid media supplemented with (+)‐GR24 or (−)‐2′‐*epi*‐GR24 (10^−4^ M), and an acetone control in parallel were replaced in each flask, and after 48 h, the mycelia were collected. To evaluate the expression of genes putatively involved in carotenoid biosynthesis and cleavage, the WT was grown as described before for 7 d. Then, the liquid media supplemented with GR24 (10^−4^ M) or acetone were put in each flask, and after 48 h, the mycelia were collected.

Total RNA was extracted using the Plant RNeasy Kit (Qiagen) and treated with TURBO™ DNase (Ambion) according to the manufacturer’s instructions. cDNA synthesis was carried out on total RNA using Super‐ScriptII (Invitrogen). RT‐qPCR experiments were carried out using a Rotor Gene apparatus (Qiagen) as described in Volpe *et al*. (2020) with two technical replicates. The comparative threshold cycle method (Rasmussen, 2001) was used to calculate relative expression levels, with *C. parasitica CpGAPDH* and *CpTubulin* as reference genes. Oligonucleotide sequences are listed in Table S1.

### Virulence assay

*CpD14* knockout mutant ability to induce canker on European chestnut (*Castanea sativa*) barks was assayed on dormant cuttings as previously described in Rostagno *et al*. (2010). Lesions were measured at 30 d post‐inoculation (dpi).

## Results

### Characterization of CpD14

The sensitivity of *C. parasitica* to the four GR24 stereoisomers was evaluated using the *in vitro* growth assay previously described (Belmondo *et al*., 2017). At 10^−4^ M, the stereoisomers whose stereochemistry corresponds to natural canonical SLs, (+)‐GR24 (strigol‐type) and (−)‐2′‐*epi*‐GR24 (orobanchol‐type), which have the D‐ring with 2′*R* configuration, and the (−)‐GR24 stereoisomer (2′*S* configuration), whose stereochemistry is not encountered in natural SLs, led to a reduction of fungal colony diameter at 72 and 96 h after inoculation (Fig. S1a). At 10^−5^ M, only the two molecules corresponding to natural SL stereochemistry, (+)‐GR24 and (−)‐2′‐*epi*‐GR24, led to a transient decrease of radial growth (Fig. S1b). *Cryphonectria parasitica* seems therefore more sensitive to GR24 stereoisomers whose stereochemistry corresponds to natural SLs.

**Fig. 1:**
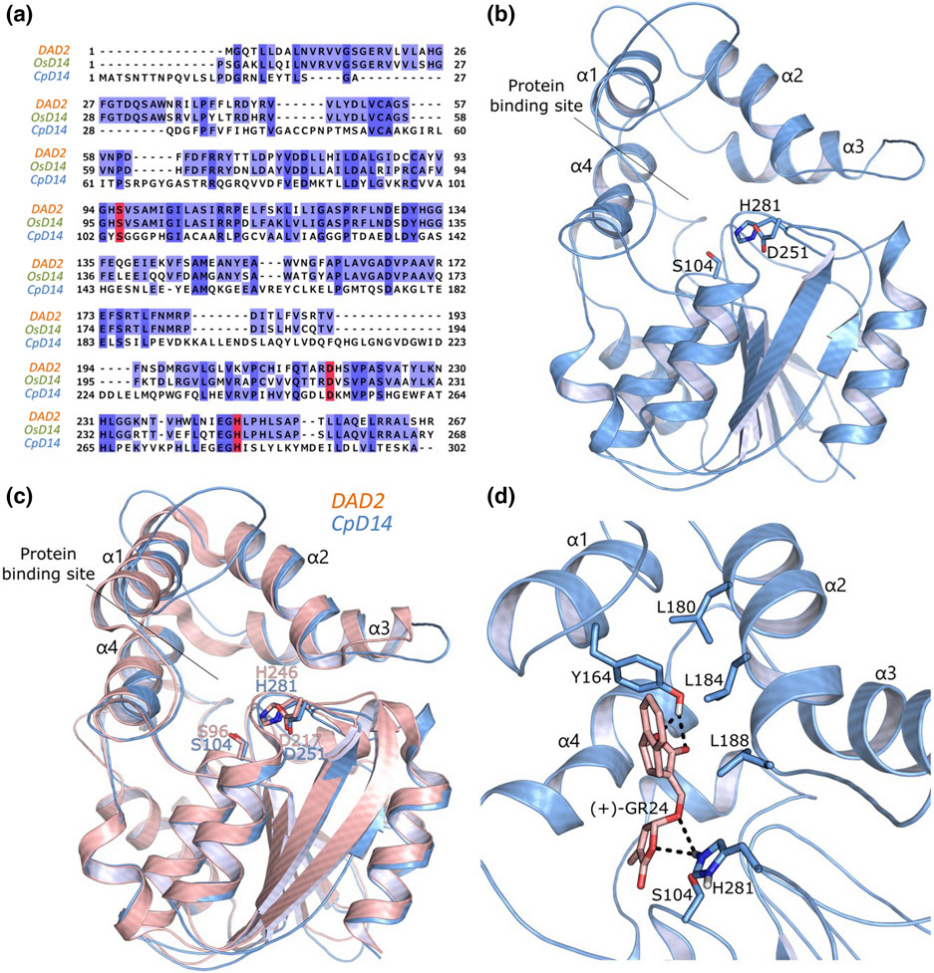
Molecular homology modelling and docking of CpD14. (a) Sequence alignment of DAD2, OsD14 and CpD14. The red boxes indicate the catalytic residues. (b) Homology model of CpD14 shown as light blue cartoon. The catalytic residues are shown as capped sticks and labelled. (c) Structural alignment of the CpD14 model (light blue) and the DAD2 template (light pink; Protein Data Bank code: 4ndp). Helices α1–α4 are labelled. The catalytic residues (Ser, Asp and His: SDH) are labelled and shown as capped sticks in the inset. (d) Docking pose of (+)‐GR24 in the CpD14 model binding site. The compound and some of the residues defining the binding site are shown as capped sticks and labelled. Hydrogen bonds are represented as dashed lines.

To identify candidate D14 homologues in the genome of *C. parasitica* (Crouch *et al*., 2020), a Blastp search, using the rice OsD14 sequence as a query, was performed under low stringency conditions (Expected value 10). We identified a sequence of 302 amino acids, hereafter called CpD14, showing 26% identity with rice D14 and 25% with the petunia homologue DAD2, and 66% coverage. The putative catalytic triad, formed by serine, aspartate and histidine (Ser‐S, Asp‐D and His‐H), and needed for SL hydrolysis (Hamiaux *et al*., 2012), is conserved among the three sequences, as shown by the alignment (Fig. 1a; CpD14: Ser104, Asp251, His281; OsD14: Ser97, Asp218, His247; and DAD2: Ser96, Asp217, His246).

In the absence of structural information, we built the 3D model of CpD14 by homology modelling. We first performed a similarity search within the PDB database using Blast and identified the structure of DAD2 from petunia as the best possible template (PDB code 4dnp; Hamiaux *et al*., 2012). DAD2 shares *c*. 76% sequence identity with rice OsD14. The resulting protein model revealed the canonical α/β hydrolase fold and the lid of the binding site surrounded by four α helices (α1–α4 in Fig. 1b). The superposition with the DAD2 template showed completely overlapping catalytic residues (Fig. 1c), and a highly disordered loop next to helix α. The quality validation of the predicted model revealed that 87.9% of residues were in favoured regions, 7.5% in allowed regions and 4.6% in disallowed ones (Fig. S2). The model was also submitted to 200‐ns‐long MD simulation, which further proved its stability, apart from the flexible close to helix α1.

Crystallographic evidence of the capability of SLs, in particular (+)‐GR24, to bind OsD14 has been already reported (PDB code: 5dj5; Zhao *et al*., 2015). Considering the high sequence identity shared by OsD14 with the DAD2 orthologue and the conservation of the general folding and the critical residues (Fig. S3a), we hypothesized that SLs would also be able to bind DAD2 and the derived CpD14‐like model. We, thus, performed molecular docking simulations of (+)‐GR24, (−)‐2′‐*epi*‐GR24 and (+)‐strigol in the CpD14 model binding site (Figs 1d, S3c,d). (+)‐GR24 assumes a conformation similar to that in OsD14 (Fig. S3b). The D‐ring faces the catalytic residues and H‐bonds, His281, the polar head of the ABC‐ring system H‐bonds to Tyr164 on α1, whereas the AB‐ring system is partially exposed towards the solvent.

**Fig. 2:**
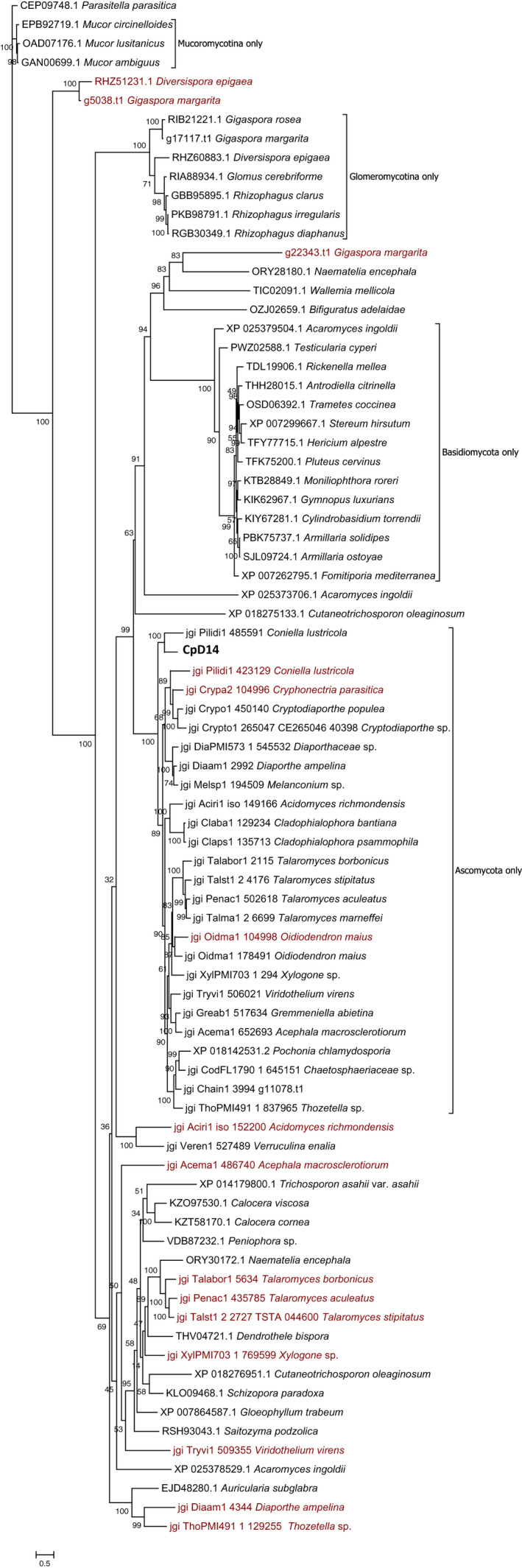
Phylogenetic analysis of putative fungal DWARF14 (D14) homologues. Through Blast analysis, several fungal D14‐like homologues conserving the SDH catalytic triad were retrieved from fungal genomes. Proteins obtained were aligned with Clustal Omega, and the obtained alignments were used to build a phylogenetic tree using the maximum likelihood (ML) method (using IqTree webserver with automatic substitution model selection). CpD14 is shown in bold with larger font size. Tree topology allows to identify clades hosting only protein belonging to Ascomycota, Basidiomycota, Mucoromycota and Glomeromycota, while other clades host proteins from fungi belonging to different taxonomical groups. For some fungi, two α/β hydrolases conserving the Ser/His/Asp catalytic triad could be found. The second Blast hit in each genome (and less conserved compared to CpD14) is given in red.

We next assessed the occurrence of CpD14 homologues in the fungal kingdom and attempted to trace their evolutionary history. We searched a number of representative fungal genomes of the Dikarya and Mucoromycota groups by Blastp using CpD14 as query and retrieved the first hit. Furthermore, given that the *C. parasitica* genome contains a second protein with similarity to CpD14, we repeated a Blastp search using, within each species, the first retrieved protein sequence as a query. All these protein sequences were aligned by Clustal Omega, and a phylogenetic tree was reconstructed using the maximum‐likelihood method (Fig. 2). Notably, we could identify α/β hydrolase proteins showing the conserved SDH catalytic triad in both Dikarya and Mucoromycota. Nevertheless, homologues of this protein cannot be found in some fungal lineages such as the vast majority of Saccharomycotina and in some well‐studied fungal model systems, that is *Neurospora crassa*, *Magnaporthe oryzae*, *Blumeria graminis* and *Ustilago maydis*. In *Aspergillus flavus,* the homologue is present, but it lacks the His residue in the catalytic domain. The closest homologues to CpD14 from each fungal genome (indicated in black in Fig. 2) cluster in three well‐supported clades, each only hosting proteins from Mucoromycota, Basidiomycota or Ascomycota. On the contrary, most of the proteins identified through the second Blastp search in each specific genome sequence (in red in Fig. 2) are scattered in mixed branches with proteins from Basidiomycota. The only exceptions are proteins from *C. parasitica*, *Coniella lustricola* and *Oidiodendron maius,* which cluster only in the Ascomycota branch together with their first hit, showing evidence of possible more recent duplication events. In summary, our analysis shows a relatively widespread occurrence in fungal genomes of at least one putative homologue of CpD14 with a conserved catalytic domain (SDH catalytic triad). Phylogenetic reconstruction of these homologues shows some correlations with taxonomic groups. A second protein, sharing some similarity with fungal D14L‐like proteins, is often present within single genomes, but with no clear association with taxonomically uniform groups.

As a further characterization of the *CpD14* gene, we searched for evidence of *CpD14* expression in the free‐living mycelium and of possible regulation by a 48‐h exposure to a GR24 racemic solution. *CpD14* was expressed in the fungus grown in liquid culture, and no difference was observed upon GR24 treatment (Fig. S4a).

Taken as a whole, these data indicate the occurrence of putative homologues of plant D14 in fungi.

### CpD14 binds and hydrolyses SLs

**Fig. 3:**
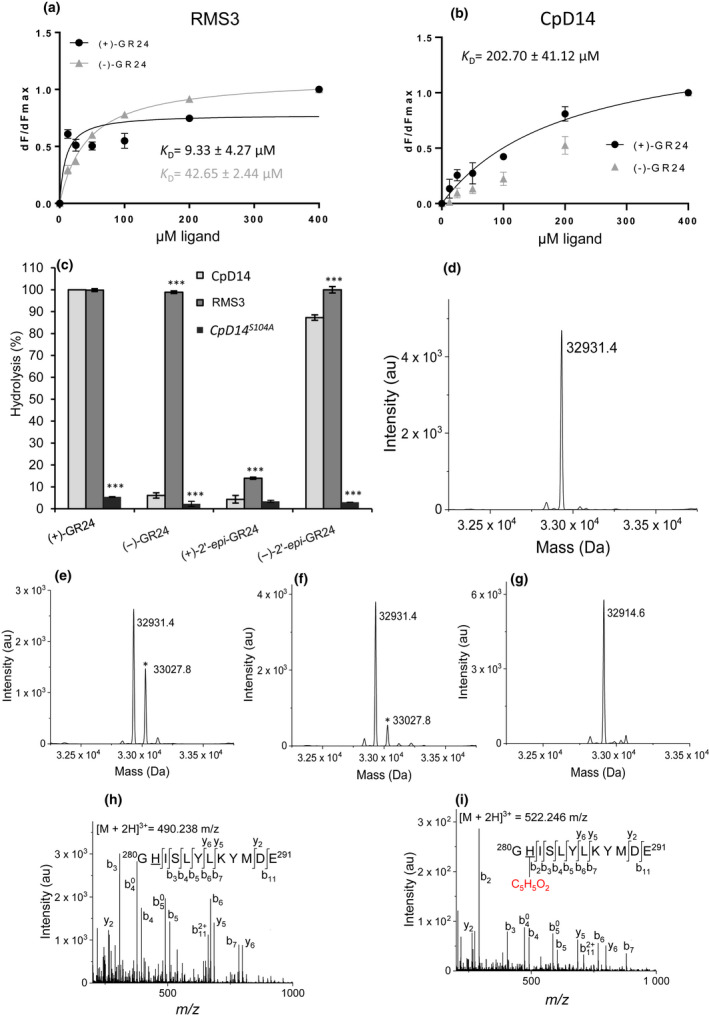
Biochemical characterization of CpD14. Biochemical analysis of the interaction between the RMS3/PsD14 and CpD14 proteins and GR24 isomers based on intrinsic tryptophan fluorescence (a, b). Plots of fluorescence intensity vs strigolactones (SLs) concentrations. The change in intrinsic fluorescence of RMS3 (a) and CpD14 (b) was monitored and used to determine the apparent *K*_D_ values. The plots represent the mean of two replicates, and the experiments were repeated at least three times. Error bars represent the SD of three replicates (means ± SD, *n* = 3). The analysis was performed with GraphPad Prism 8.0 Software. CpD14 enzymatic activity towards GR24 isomers (c). (+)‐GR24, (−)‐GR24, (+)‐2′‐*epi*‐GR24 and (−)‐2′‐*epi*‐GR24 at 10 µM were incubated with RMS3, CpD14 and CpD14^S104^ at 5 µM for 150 min at 25°C. UPLC‐UV (260 nm) was used to detect the remaining amount of GR24 isomers. Columns represent the mean value of the hydrolysis rate calculated from the remaining GR24 isomers, quantified in comparison with (±)‐1‐indanol, as internal standard. Error bars represent the SD of three replicates (means ± SD, *n* = 3). The asterisks indicate statistical significance from the CpD14 protein sample: ***, *P* ≤ 0.001 (as measured by Kruskal–Wallis test). Mass spectrometry characterization of covalent CpD14–ligand complexes. Deconvoluted electrospray mass spectra of CpD14 before (d) and after adding (+)‐GR24 (e) or (−)‐GR24 (f). Peaks with an asterisk correspond to proteins covalently bound to a ligand. The mass increments are measured for different protein–ligand complexes: 96.4 Da for (+)‐GR24) and (−)‐GR24). Deconvoluted electrospray mass spectra of CpD14^S104A^ (g). Fragmentation spectra of unmodified (h) and ligand‐modified peptide (i). Labelled peaks correspond to b and y fragments of the double‐charged precursor ion displayed on the top. The His residue (H281) modified by the ligand is underlined on the sequence. In red, formula of the adduct determined by MS.

We studied the putative interaction of the CpD14 protein with SLs by expressing and purifying the CpD14 protein *in vitro* and assessing its ability to interact with GR24 isomers (Fig. 3). The interaction between (+)‐GR24 and CpD14 was confirmed using tryptophan intrinsic fluorescence assay (Figs 3a,b, S5), even if a lower affinity was observed in comparison with RMS3/PsD14 (*K*_D_ = 202.70 ± 41.12 µM vs 9.33 ± 4.27 µM), the SL receptor described in pea (de Saint Germain *et al*., 2016). To confirm this interaction, we used nano‐differential scanning fluorimetry (nanoDSF) recording changes in tryptophan fluorescence (ratio 350 nm : 330 nm) during protein denaturation. We highlighted interactions by noticing a change of initial fluorescence ratio, but we failed to determine the CpD14 melting temperature due to the intrinsic nature of the protein. Therefore, this technique did not allow us to determine potential CpD14 conformational changes as it was done with RMS3, where (+)‐GR24 (strigol‐type) and (−)‐2′‐*epi*‐GR24 (orobanchol‐type) induce a larger destabilization in comparison with (−)‐GR24 and (+)‐2′‐*epi*‐GR24, whose stereochemistry is not encountered in natural SLs. We detected no change in the CpD14 melting temperature (Fig. S5) with the four GR24 isomers.

As a further step, by using the internal standard (±)‐1‐indanol, the cleavage activity of the CpD14 towards (+)‐GR24, (−)‐GR24, (+)‐2′‐*epi*‐GR24 and (−)‐2′‐*epi*‐GR24 was quantified and compared to the cleavage activity of RMS3 and CpD14^S104A^, a CpD14 sequence variant where the serine at position 104 of the putative SDH catalytic triad was replaced with an alanine. CpD14 efficiently cleaved (+)‐GR24 and (−)‐2′‐*epi*‐GR24, like RMS3, but poorly (−)‐GR24 and (+)‐2′‐*epi*‐GR24 (Fig. 3c). No significant cleavage activity was recorded for CpD14^S104A^ with any of the GR24 isomers tested (Fig. 3c). These data highlight that CpD14 hydrolyses GR24 isomers with the configuration of natural SLs, and that this hydrolysis cannot occur without an intact catalytic triad. GC profluorescent probes, which were used to determine RMS3 kinetics (de Saint Germain *et al*., 2016, 2021), were poorly hydrolysed by CpD14 (Fig. S6). However, after incubating (+)‐GR24 and (−)‐GR24 with CpD14 and recording mass spectrometry spectra in denaturing conditions, we detected in all cases a mass shift corresponding to the formation of a covalent complex, with the D‐ring (Fig. 3d–i). According to the hydrolysis results (Fig. 3c), the formation of the covalent complex was favoured with (+)‐GR24 (Fig. 3e) in comparison with (−)‐GR24 (Fig. 3f), while no complex was observed for CpD14^S104A^ (Fig. 3g). Fragmentation spectra of unmodified and ligand‐modified peptides unambiguously supported the D‐ring being specifically attached to His281 of the catalytic triad (Fig. 3h,i). We therefore demonstrated that CpD14 forms a stable intermediate with the D‐ring, as previously reported for other SL receptors (PsD14/RMS3, AtD14, D14 and ShHTL7 and PrKARRIKIN INSENSITIVE2d3 (PrKAI2d3), the putative orthologues in *Striga hermontica* and *Phelipanche ramosa*, respectively) (de Saint Germain *et al*., 2016, 2021; Yao *et al*., 2016, 2017, 2018a,b).

As a further step, we evaluated whether CpD14 could complement the phenotypic defects of the *A*. *thaliana d14‐1* mutant line. Three transgenic lines (#3.2, #7.5 and #9) were generated expressing the *CpD14* cDNA as a 6X‐HA‐tagged fusion protein under the *A. thaliana D14* promoter (Fig. S7a). The shoot branching (Fig. S7b,c) and plant height (Fig. S7b,c) phenotypes of the *Atd14‐1* mutant were not rescued by *CpD14* expression, as by contrast, it was observed in the line transformed with the *AtD14* gene, which was used as a positive control (Fig. S7b,c). It is worth noting that, even if expressed under the same promoter, CpD14 was much less abundant in Arabidopsis leaves than AtD14 (Fig. S7a), suggesting a different stability or turnover of CpD14 compared to AtD14 or a lower expression due to lack of codon usage optimization between fungal coding and plant coding D14 homologues.

Although the complementation assay was not successful, the biochemical characterization of the protein clearly shows that CpD14 binds and hydrolyses GR24 natural stereoisomers thanks to the SDH catalytic triad.

### *In vitro* fungal development upon GR24 treatment is altered in strains defective of *CpD14*

**Fig. 4:**
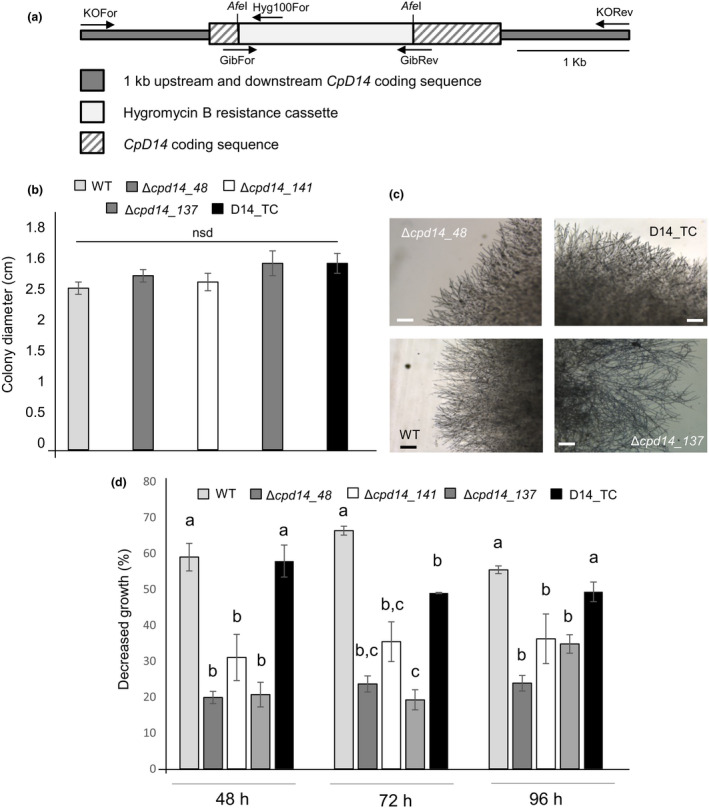
Characterization of the *CpD14* knockout mutants. (a) Scheme of the knockout construct. CpD14 knockout mutants were obtained through double homologous recombination with a construct carrying a hygromycin B resistance cassette interrupting the gene sequence. Around 1 kb upstream and downstream sequences of the *CpD14* gene were used to induce homologous recombination. Primers used for the screening of transformed colonies are shown. (b, c) Colony growth pattern of *Cryphonectria parasitica* wild‐type (WT) and Δ*cpd14* mutants grown under normal condition. *Cryphonectria parasitica* WT, Δ*cpd14* mutants (Δ*cpd14_48*; Δ*cpD14_141*; Δ*cpD14_137*) and negative transformation control (D14_TC) strains were grown on B5 solid medium + 2% glucose at 20°C in the dark. Histograms represent the colony growth diameter of each strain after 48, 72 and 96 h of inoculation. Each strain was analysed in triplicate. Data for each condition are presented as mean ± SE. nsd, no statistically significant difference. (c) Pictures taken with a stereomicroscope at the edge of the colony, 96 h post‐inoculation. Bars, 100 μm. (d) Effect of (+)‐GR24 on the growth of *C. parasitica* WT and Δ*cpd14* mutants. *Cryphonectria parasitica* WT, Δ*cpd14* mutants (Δ*cpd14_48*; Δ*cpD14_141*; Δ*cpD14_137*) and negative transformation control (D14_TC) strains were grown on B5 solid medium (supplemented with 2% glucose) at 20 °C in the dark. Histograms represent the decreased growth (%) of each strain obtained comparing strain colony diameter upon (+)‐GR24 treatment with that measured upon mock treatment (acetone) 48, 72 and 96 h after inoculation. Each strain was analysed in triplicate. Data for each condition are presented as mean ± SE. Different letters indicate statistically significant difference (*P* < 0.05, ANOVA) within each time point.

Three independent *C. parasitica* knockout mutants (Δ*cpd14_48*, *Δcpd14_137* and Δ*cpd14_141*) were generated using site‐directed double homologous recombination (Fig. 4a). Ninety‐six hours post‐inoculation, the *in vitro* growth of the mutant strains under standard conditions was comparable to that of the WT strain (Fig. 4b,c). The same behaviour was shown by a control strain (D14_TC), which had been subjected to the process of genetic transformation but without the construct leading to the specific mutation on *CpD14* gene (Fig. 4b,c).

**Fig. 5:**
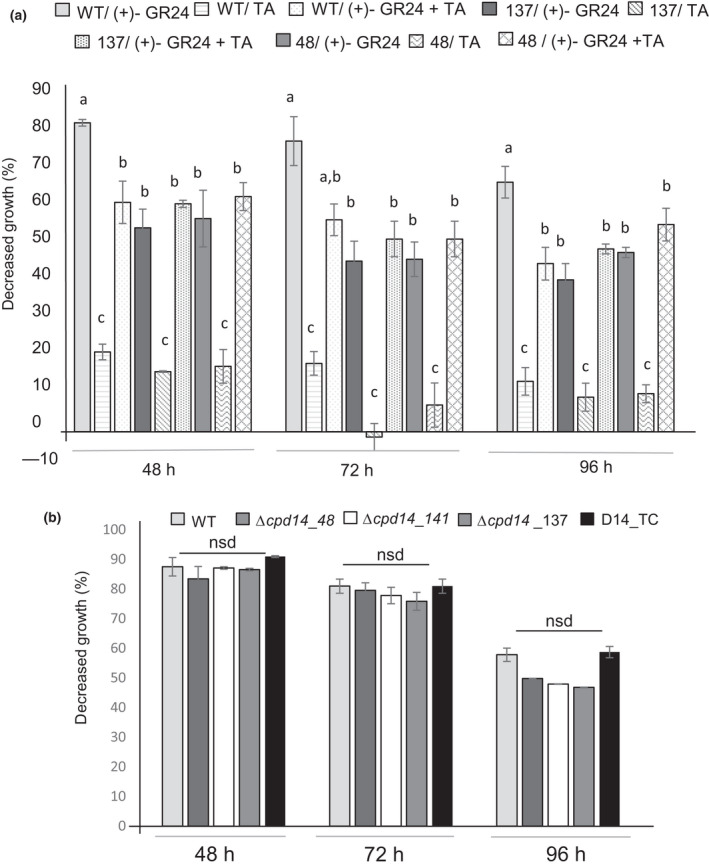
Effect of tolfenamic acid (TA) and zaxinone treatments on *Cryphonectria parasitica* wild‐type (WT) and Δ*cpd14* mutants. (a) *Cryphonectria parasitica* WT and Δ*cpd14* mutants (Δ*cpd14_48*; Δ*cpD14_141*; Δ*cpD14_137*) strains were grown on B5 solid medium (supplemented with 2% glucose) at 20°C in the dark. Histograms represent the decreased growth (%) of each strain obtained comparing colony diameter upon (+)‐GR24, TA, a strigolactone (SL) receptor inhibitor (Hamiaux *et al*., 2018) and TA + (+)‐GR24 treatments with the colony diameter measured upon the corresponding mock treatment (acetone, dimethyl sulfoxide (DMSO) and DMSO + acetone, respectively) 48, 72 and 96 h after inoculation. Each molecule was added once in the media: (+)‐GR24 had a final concentration of 10^−4^ M, while TA had a final concentration of 10^−6^ M. (b) Effect of zaxinone treatments on *C. parasitica* WT and Δ*cpd14* mutants. *Cryphonectria parasitica* WT, Δ*cpd14* mutants (Δ*cpd14_48*; Δ*cpD14_141*; Δ*cpD14_137*) and negative transformation control (D14_TC) strains were grown on B5 solid medium (supplemented with 2% glucose) at 20°C in the dark. Histograms represent the decreased growth (%) of each strain obtained comparing colony diameter upon zaxinone (Wang *et al*., 2019) treatment with that measured upon mock treatment (acetone) 48, 72 and 96 h after inoculation. Each strain was analysed in triplicate. Data for each condition are presented as mean ± SE. Different letters indicate statistically significant differences (*P* < 0.05, ANOVA) within each time point. nsd, no statistically significant difference.

The effect of SLs on the mutant strains was then tested by considering an exposure to (+)‐GR24. Notably, the *in vitro* growth of the three mutants was less inhibited compared to that of the WT or the control strain D14_TC (Fig. 4d). We also tested the effect of tolfenamic acid, which has been recently described as an inhibitor of plant SLs receptors (Hamiaux *et al*., 2018). The tolfenamic acid treatment slightly reduced the *in vitro* fungal growth of WT and mutant strains but, notably, the treatment decreased the growth inhibition induced by GR24 in the WT strain (Fig. 5a), confirming its inhibitory activity towards GR24 perception also in fungi. Interestingly, the tolfenamic acid treatment did not influence the growth of Δ*cpd14* knockout mutants upon (+)‐GR24 treatment (Fig. 5a) further confirming *in vivo* interaction between GR24 and CpD14.

To get further insights on the link between CpD14 and GR24 effect on fungal growth, we analysed the effect of zaxinone, another carotenoid‐derived molecule which we previously described as a plant growth regulator also involved in the establishment of the AM symbiosis, as a rice line defective of the gene responsible for zaxinone synthesis showed a reduced mycorrhiza formation (Wang *et al*., 2019). We investigated the effect of zaxinone on WT and Δ*cpd14*‐mutant strains. Zaxinone exposure led to a strong growth inhibition of the WT strain and the same effect was observed in the Δ*cpd14* mutants (Fig. 5b), indicating that CpD14 is not involved in the response to another signalling apocarotenoid.

To investigate the putative involvement of CpD14 in the fungus–plant interaction, we performed a virulence assay. Wild‐type and Δ*cpd14*‐mutant strains were inoculated on chestnut cuttings, and lesions were measured after 30 d. Under this condition, no difference was observed between WT and mutant strains (Fig. S8).

We also hypothesized that CpD14 could have an endogenous role; we thus investigated in *C. parasitica* the occurrence of genes putatively involved in carotenoid biosynthesis and cleavage, based on those described in the model fungus *Neurospora crassa*. We found genes encoding a putative cyclase and phytoene synthase (CpPHYSYN), a phytoene dehydrogenase (CpPHYDE), a carotenoid oxygenase (CpCARX), a neurosporaxanthin–toluene carotenoid oxygenase 2 (CpOCO) and an aldehyde dehydrogenase (CpADE). We then analysed their expression profile in the WT and the mutant strains. *CpPhySin* and *CpPhyADE*, which are placed at the beginning of the carotenoid pathway, showed a lower expression in both mutants compared to the WT strain, while no difference was detected for *CpPHYDE*, *CpCARX* and *CpOCO* (Fig. S4b–f). This result indicates that the lack of CpD14 induces a perturbation on the expression of some genes of the carotenoid pathway and suggests that CpD14 could have also an endogenous role.

## Discussion

### *In silico* studies reveal putative homologues of D14 in fungi

SLs are multifunctional plant metabolites acting as a class of hormones controlling plant developmental processes and interactions with other organisms (Lopez‐Raez *et al*., 2017; Lanfranco *et al*., 2018b; Carvalhais *et al*., 2019); they were even shown to have an impact on human cell lines as anticancer, anti‐inflammatory and antiviral drugs (Prandi *et al*., 2021). SLs have therefore multiple target organisms, which, in turn, had to evolve a molecular machinery to perceive and respond to them. The SL receptor D14, with the unique feature of coupled enzyme and receptor functions, and several downstream signalling components have been characterized in plants (Mashiguchi *et al*., 2021). So far, no SL receptor/early signalling components have been described in fungi. Unfortunately, AM fungi, which display the most studied biological response to SLs in the fungal kingdom, are not a suitable experimental system for a reverse genetic approach because of the lack of stable genetic transformation protocols. Here, we exploited *C. parasitica,* a filamentous fungus, previously shown to be sensitive to (±)‐GR24 (Belmondo *et al*., 2017) and for which genetic and genomic resources are available (Crouch *et al*., 2020), to look for candidate SLs receptors. Using the same biological assay set‐up by Belmondo *et al*. (2017), we demonstrated here that *C. parasitica* is more sensitive to GR24 isomers whose stereochemistry corresponds to natural SLs, pointing to a biological response strongly linked to specific SL stereochemistry.

A candidate protein, named CpD14, was identified by Blastp searches under low stringency conditions and characterized *in silico* using molecular homology modelling and docking. The 3D structure of CpD14, obtained using DAD2 as a template, showed the typical fold of α/β hydrolase enzymes; the alignment and superposition with petunia DAD2 and OsD14 revealed the conservation of the catalytic SDH triad. Docking simulations proved a good positioning of the ligand, correctly orienting the D‐ring to the catalytic residues. These results, further confirmed by the correspondence between the docking pose of (+)‐GR24 in CpD14 and its crystallographic pose in OsD14, support the prediction that CpD14 may interact with and hydrolyse SLs.

Our survey within fungal kingdom databases clearly shows the widespread occurrence in fungal genomes of putative homologues of CpD14 with a conserved SDH catalytic triad; this finding suggests their potential involvement in important adaptive functions. However, to our knowledge, none of these fungal genes, annotated as α/β hydrolases or hypothetical proteins, has been so far characterized through a reverse genetic approach. Phylogenetic reconstruction shows some degree of correlation with taxonomic groups. Notably, the sequences identified in genomes of AM fungi, as first hits using CpD14 as query, form a cluster of Glomeromycotina‐only sequences (Fig. 2). It would be interesting to further characterize these sequences and test their involvement in SLs response.

### CpD14 hydrolyses the natural stereoisomers of GR24 *in vitro* and is involved in the SLs‐induced fungal growth suppression *in vivo*

*In vitro* biochemical characterization of the CpD14 recombinant protein, by using the tryptophan intrinsic fluorescence assay, demonstrated that it interacts with (+)‐GR24, even if this is with a 20‐fold lower affinity compared to RMS3/PsD14. Notably, CpD14 displays an α/β hydrolytic activity *in vitro* on GR24 with a preference for isomers showing natural stereochemistry, and this enzymatic activity requires an intact catalytic triad. In addition, we demonstrated that CpD14 forms a covalent complex with the D‐ring *via* the histidine residue of the catalytic triad. This has been previously reported for plant SLs receptors such as RMS3/PsD14, AtD14, ShHTL7 and OsD14 (de Saint Germain *et al*., 2016; Yao *et al*., 2016, 2017, 2018). Thus, computational and *in vitro* biochemical analyses indicate that CpD14 is able to bind and hydrolyse SLs with a mechanism similar to the one deciphered for plant SL receptors.

To clarify the role of CpD14 in the suppression of fungal growth by GR24 observed *in vitro*, CpD14 knockout mutants were generated by homologous recombination. The mutants displayed a reduced sensitivity to (+)‐GR24 compared to the WT strain, although the response was not fully abolished. The lack of a complete insensitivity could be explained by the presence of a second gene in the *C. parasitica* genome encoding a protein with 49% identity to CpD14 that may partially complement lack of CpD14 function. The analysis of a double knockout mutant can verify this hypothesis.

The exposure to tolfenamic acid, an inhibitor of plant SL receptors (Hamiaux *et al*., 2018), reduced the effect of GR24 on the growth of the WT *C. parasitica*, suggesting a conserved inhibitory activity on CpD14. Interestingly, tolfenamic acid did not exert any effect on the Δ*cpd14* mutants. The specificity of the response to SLs was assessed by considering zaxinone, a recently described natural metabolite, which is also derived from the carotenoid pathway (Wang *et al*., 2019). Zaxinone inhibited growth in the WT strain, similar to what observed for (+)‐GR24, as well as in the Δ*cpd14* mutants. Thus, although the reasons of the inhibitory effect of zaxinone are unknown, these results clearly indicate that the response to zaxinone does not involve CpD14. Overall, these data demonstrate that *C. parasitica* growth reduction induced by (+)‐GR24 is mediated by an active CpD14, a protein able to bind and hydrolyse (+)‐GR24 *in vitro*. However, it must be noted here that CpD14 expression under the control of the *AtD14* promoter did not complement the *d14‐1* mutation in *A. thaliana*. Besides protein abundance issues (Fig. S7a), we can also hypothesize that signalling components downstream of D14 may not be properly activated in the CpD14‐complemented *A. thaliana* mutant. Indeed, by using the tryptophan intrinsic fluorescence assay, we could not verify the occurrence of CpD14 conformational changes upon SL binding, an event that is required for downstream signalling in plants (Yao *et al*., 2018).

A still‐open question is the biological role of CpD14 in *C. parasitica*. The mutants do not display an obvious phenotype under standard laboratory growth conditions, unless they are exposed to bioactive stereoisomers of synthetic SLs. We hypothesized a theoretical interaction with plant‐derived SLs in chestnut bark, the site of entry and initial pathogenic interaction in *C. parasitica*—chestnut. However, no differences were found between WT and mutant strains in a pathogenicity test performed on chestnut cuttings, which allows for the measurement of initial pathogenicity and virulence potential of the fungus. By contrast, in infections occurring in nature, a typical secondary symptom of *C. parasitica* infection is epicormic bud outgrowths around the canker region of the bark lesion, particularly in cankers with an intensive lethal infection outcome (Rigling & Prospero, 2018). In this regard, although no specific data are available for the presence of SLs and their role in chestnut trees, we can speculate that such bud outgrowths may be linked to SLs regulation mediated by *C. parasitica* infection. Indeed, SLs were shown to be negative regulators of axillary bud outgrowth (Ruyter‐Spira *et al*., 2013), an activity that is maintained in the model tree poplar (Muhr *et al*., 2016).

On the other hand, we can also speculate that *C. parasitica* uses CpD14 to hydrolyse endogenous carotenoid‐derived molecules, which may be important for fungal metabolism (Avalos & Limón, 2015). A survey of the *C. parasitica* genome allowed us to indeed identify several putative homologues of those genes involved in carotenoid and apocarotenoid metabolism, well described in the model fungus *Neurospora crassa*. Notably, these genes are expressed in the free‐living mycelium of *C. parasitica,* and some were found to be de‐regulated in the mutant strains. Comparing the metabolome and, notably, the apocarotenoids profile of the WT with Δ*cpd14 C. parasitica*, may provide further insights on this intriguing possibility.

In conclusion, we have characterized a fungal gene encoding a member of the α/β hydrolase fold family of enzymes. *In silico* modelling, docking and *in vitro* assays demonstrated its ability to bind and hydrolyse natural GR24 stereoisomers. The characterization of knockout fungal strains showed that the gene mediates fungal growth inhibition induced by SLs under controlled growth conditions. As a whole, our findings support the idea that SLs are multifunctional molecules acting in plant–microbe interactions. Their divergent roles in promoting root symbiosis and influencing the growth of detrimental fungi may open new perspectives for agro‐biotechnological applications.

## Author contributions

LL, VF and MT designed the investigation. VF and MF conducted experiments on growth assays and mutant generation and analyses; AdSG, DC, PLB and F‐DB were involved in the biochemical assays and Arabidopsis complementation, and GD’A and FS performed modelling and docking analyses. LL, MT, F‐DB, FS, CP, FC and SA‐B contributed to results discussion and wrote the manuscript. VF, MF and AdSG contributed equally to this work.

## Supporting information


**Fig. S1** Effect of the four GR24 stereoisomers on *Cryphonectria parasitica* growth.
**Fig. S2** Ramachandran plot of the CpD14 model.
**Fig. S3** Modelling and docking of CpD14 with DAD2 and OsD14.
**Fig. S4** Expression level of *CpD14* and genes putatively involved in carotenoid biosynthesis and cleavage in *Cryphonectria parasitica* mycelia.
**Fig. S5** Intrinsic tryptophan fluorescence of RMS3 and CpD14 proteins in the presence of SL analogues and thermostability analysed by nanoDSF.
**Fig. S6** Structures of SL profluorescent probes, and enzymatic kinetics for CpD14 (1 µM) and RMS3 (0.33 µM).
**Fig. S7** Complementation assay in *Arabidopsis thaliana atd14‐1* mutant line.
**Fig. S8** *Cryphonectria parasitica* virulence assay on chestnut cuttings.

**Table S1** List of primers.
Please note: Wiley Blackwell are not responsible for the content or functionality of any Supporting Information supplied by the authors. Any queries (other than missing material) should be directed to the *New Phytologist* Central Office.

## Data Availability

The data that support the findings of this study are available from the corresponding author upon reasonable request.
