# Supplementary material for: A structural homologue of the plant receptor D14 mediates responses to strigolactones in the fungal phytopathogen Cryphonectria parasitica
Source: New Phytol. 2022 Feb 26;234(3):1003–17. doi: 10.1111/nph.18013 (PMC9306968; doi:10.1111/nph.18013)
Supplement: Supplementary file 1 — Fig. S1 Effect of the four GR24 stereoisomers on Cryphonectria parasitica growth. Fig. S2 Ramachandran plot of the CpD14 model. Fig. S3 Modelling and docking of CpD14 with DAD2 and OsD14. Fig. S4 Expression level of CpD14 and genes putatively involved in carotenoid biosynthesis and cleavage in Cryphonectria parasitica mycelia. Fig. S5 Intrinsic tryptophan fluorescence of RMS3 and CpD14 proteins in the presence of SL analogues and thermostability analysed by nanoDSF. Fig. S6 Structures of SL profluorescent probes, and enzymatic kinetics for CpD14 (1 µM) and RMS3 (0.33 µM). Fig. S7 Complementation assay in Arabidopsis thaliana atd14‐1 mutant line. Fig. S8 Cryphonectria parasitica virulence assay on chestnut cuttings. [file NPH-234-1003-s002.pdf]

New Phytologist Supporting Information

Article title:**A structural homologue of the plant receptor D14 mediates responses to strigolactones in the fungal phytopathogen *Cryphonectria parasitica***

Authors: Valentina Fiorilli[1*], Marco Forgia[2*], Alexandre de Saint Germain[3*], Giulia D'Arrigo[4#], David Cornu[5], Philippe Le Bris[3], Salim Al-Babili[6], Francesca Cardinale[7], Cristina Prandi[8], Francesca Spyrakis[4], François-Didier Boyer[9], Massimo Turina[2], Luisa Lanfranco[1]

Article acceptance date: 26 January 2021

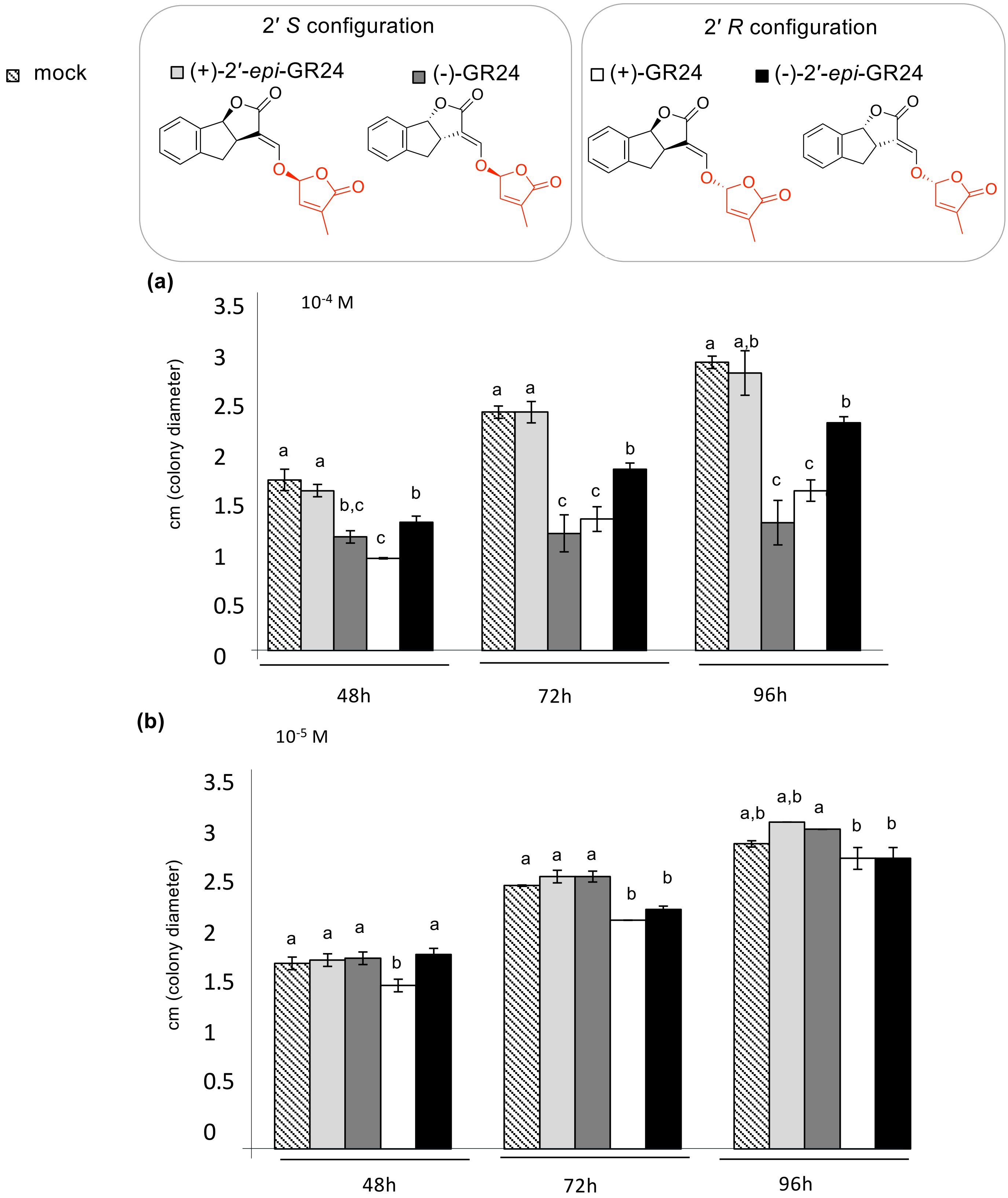

Figure S1. Effect of the four GR24 stereoisomers on *Cryphonectria parasitica* growth. *C. parasitica* WT strain was grown on B5 solid medium (supplemented with 2% glucose) at 20°C in the dark. Histograms show the colony diameter upon single stereoisomer [(+)-2'-*epi*-GR24; (-)-GR24; (+)-GR24; (-)-2'-*epi*-GR24] treatment or mock treatment (acetone) 48, 72 and 96 h after inoculation. **(a)** Colony diameter of *C. parasitica* WT strain upon the different GR24 stereoisomers treatments. Each molecule was applied individually in the media at the final concentration of 10^{-4} M. **(b)** Colony diameter of *C. parasitica* WT strain upon treatment with the different GR24 stereoisomers. Each molecule was applied individually in the media at the final concentration of 10^{-5} M. Each strain was analysed in triplicate. Data for each condition are presented as mean ± SE. Different letters indicate statistically significant differences ($p<0.05$, ANOVA) within each time point. The experiment was repeated twice with similar results. The structures of GR24 isomers are shown on top of the figure (the D ring is highlighted in red).

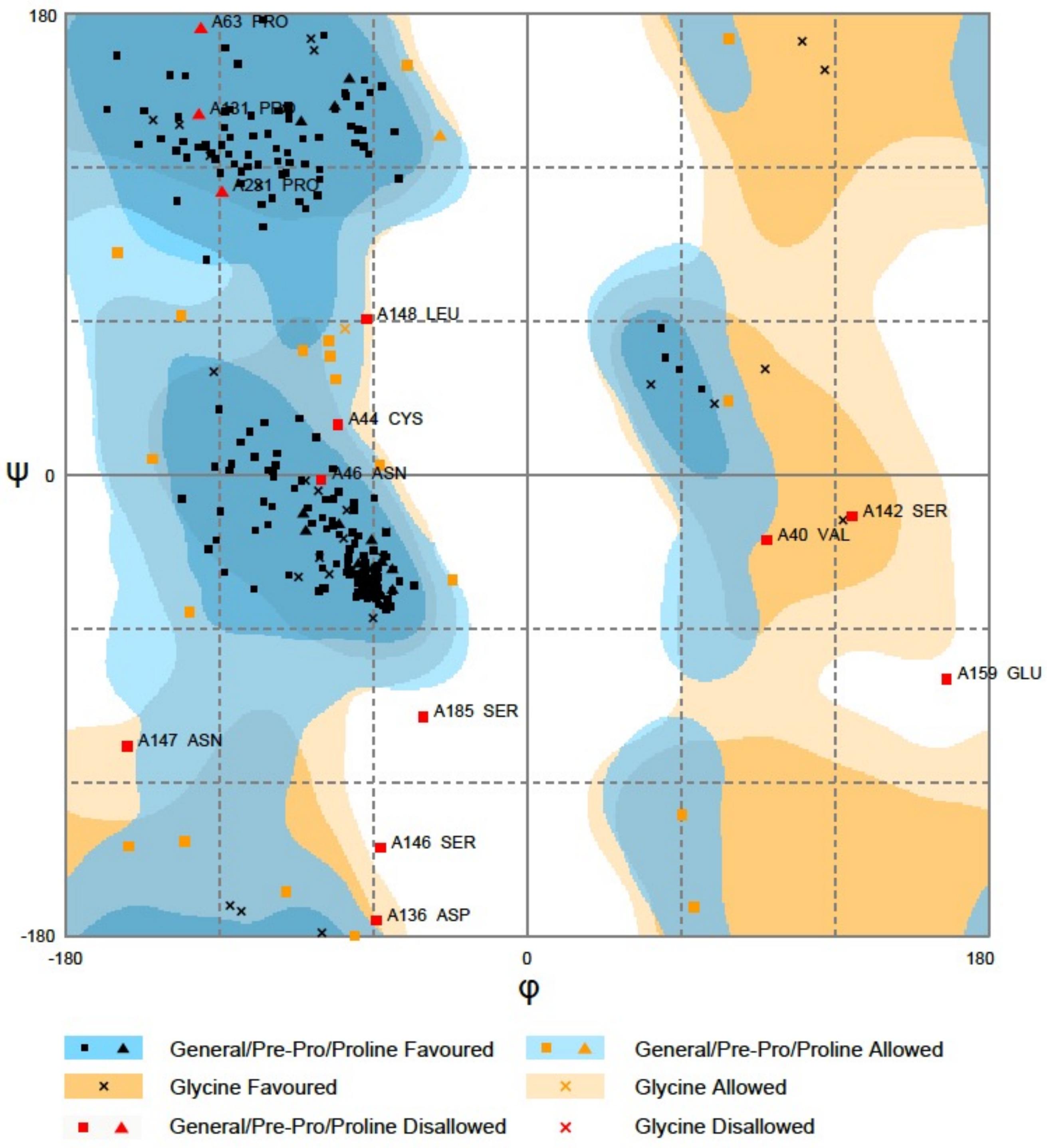

Number of residues in favoured region (~98.0% expected) : 247 (87.9%)
Number of residues in allowed region (~2.0% expected) : 21 (7.5%)
Number of residues in outlier region : 13 (4.6%)

Figure S2. Ramachandran plot of the CpD14 model.

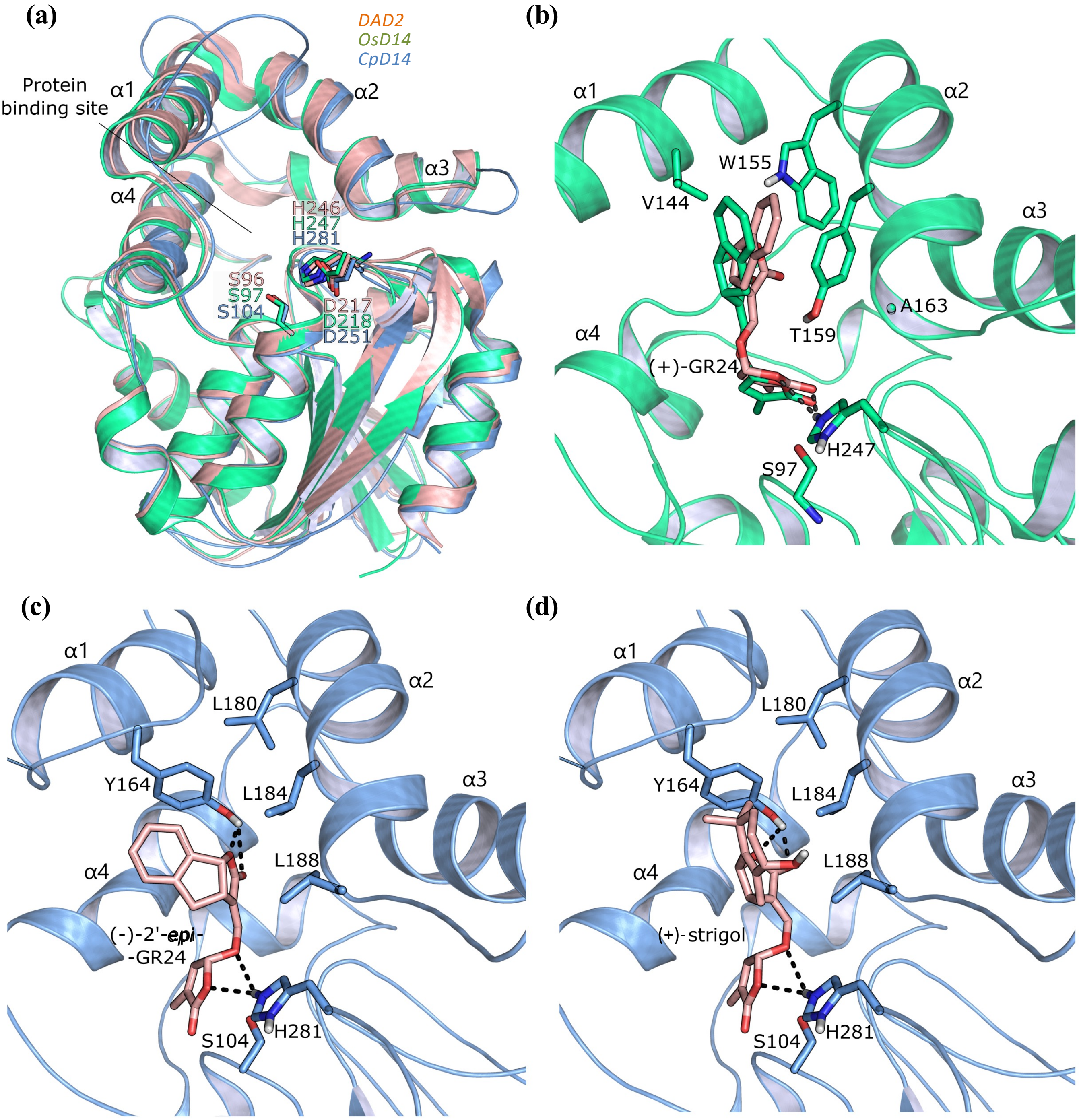

Figure S3. Modelling and docking of CpD14 with DAD2 and OsD14 (a) Structural alignment of CpD14 model with DAD2 and OsD14 colored in light blue, light pink and light green, respectively. **(b)** Superposition of the docking pose (pink) of (+)-GR24 and the crystallographic pose (green) in OsD14 X-ray structure (PDB: 5DJ5). **(c, d).** Docking pose of (-)-2'-epi-GR24 and (+)-strigol in the CpD14-like binding site, respectively**.** The compounds and some of the residues defining the binding site are shown as capped sticks and labelled. Hydrogen bonds are represented as dashed lines.

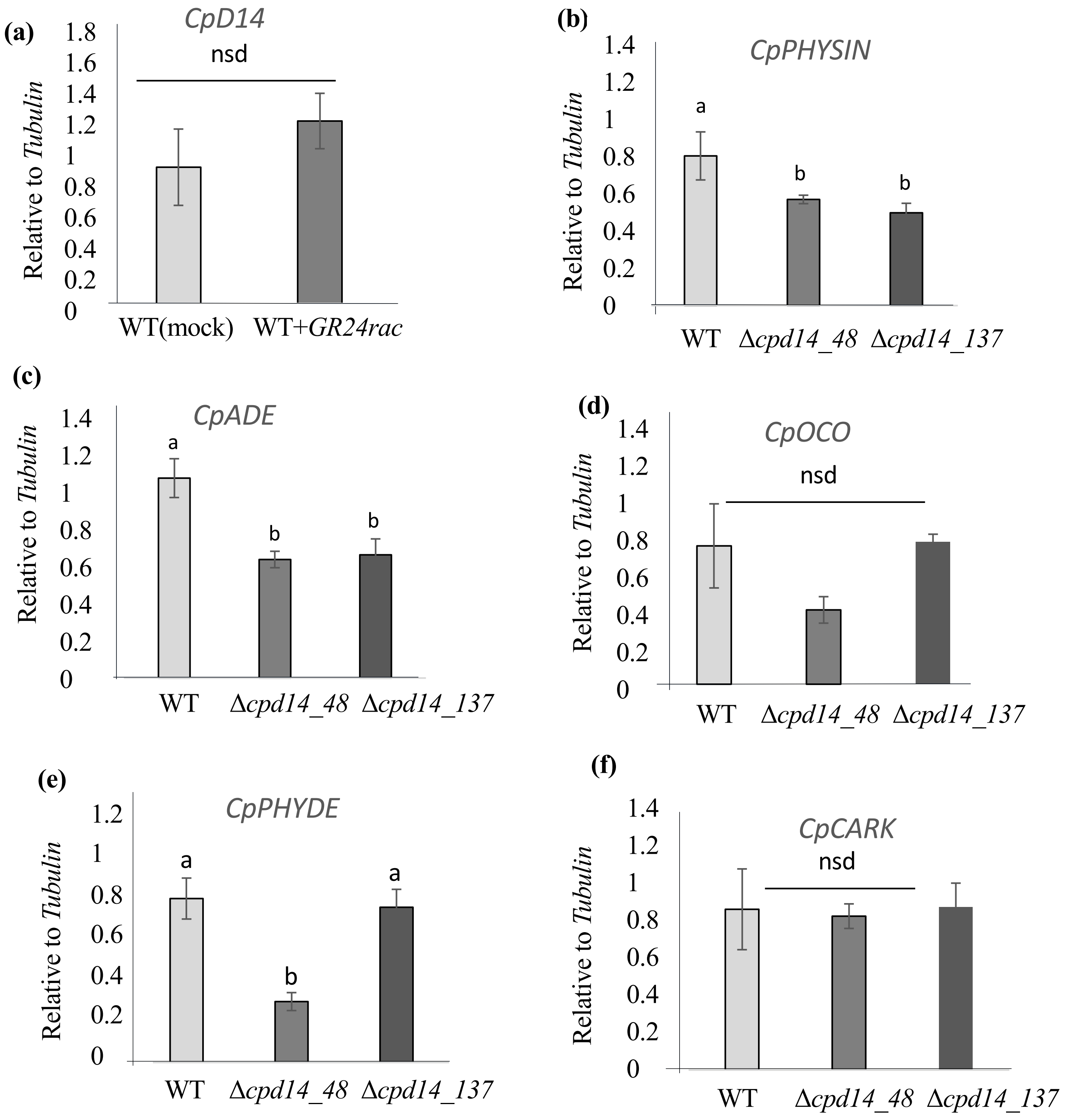

Figure S4. Expression level of CpD14 and genes putatively involved in carotenoid biosynthesis and cleavage in *Cryphonectria parasitica* mycelia. (a) Expression of *CpD14* (relative to *CpTubulin*) assessed by RT-qPCR in *C. parasitica* mycelia grown in the presence of acetone (mock treatment) or the GR24rac solution (n = 5). Data for each condition are presented as mean ± SE. No statistical differences (nsd) were detected through one-way ANOVA and Tukey's post hoc test between different treatments ($p<0.05$). (b-f) Transcripts of genes (relative to *CpTubulin*) assessed by RT-qPCR in *C. parasitica* mycelia under acetone (mock treatment) (n = 5). Data for each condition are presented as mean ± SE. Letters represent statistical differences detected through one-way ANOVA and Tukey's post hoc test between different treatments ($p<0.05$). *CpPhysyn*: KAF3768751.1 cyclase and phytoene synthase activities; *CpADE*: KAF3760879.1 aldehyde dehydrogenase; *CpOCO*: MU032354.1 neurosporaxanthin - torluene carotenoid oxygenase 2; *CpPhyde*: KAF3768752.1 Phytoene dehydrogenase; *CpCARK*: KAF3759923.1 carotenoid oxygenase.

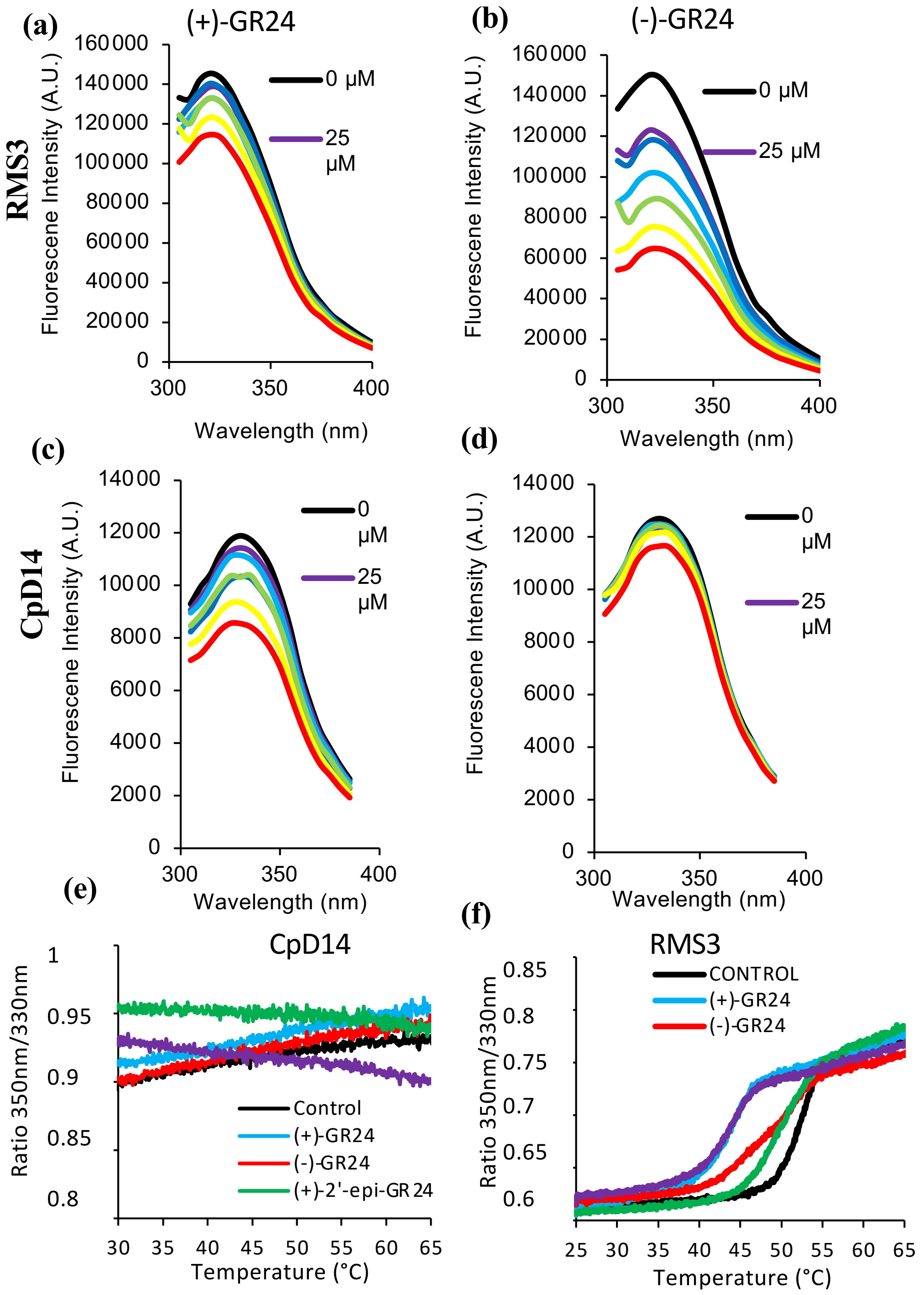

Figure S5. Intrinsic tryptophan fluorescence of RMS3 (a, b, f) and CpD14 (c, d, e) proteins in the presence of SL analogues and thermostability analysed by nanoDSF. Changes in intrinsic fluorescence emission spectra of proteins, in the presence of various concentrations of (+)-GR24 (**a**, **c**), (-)-GR24 (**b**, **d**). Proteins (10 µM) were incubated with increasing amounts of ligand (0–800 µM, top line to bottom line, respectively). The observed relative changes in intrinsic fluorescence were plotted as a function of SL analogue concentration and transformed to degree of saturation and used to determine the apparent KD values relevant to Fig. 2 (a, b). The plots represent the mean of two replicates and the experiments were repeated at least three times. The analysis was performed with GraphPad Prism 8.0 Software. (**e, f**) Results obtained with CpD14 (**e**) and RMS3 (**f**) proteins at 10 µM in the absence of ligand (black line) or the presence of various ligands at 100 µM (+)-GR24 (green line), (-)-GR24 (red line), (+)-2'-*epi*-GR24 (purple line) and (-)-2'-*epi*-GR24 (blue line). Curves show the changes in fluorescence (ratio F_{350nm}/F_{330nm}) with temperature, from which the apparent melting temperatures (T_m) for each sample were determined, when possible. The experiment was carried out twice.

(a)

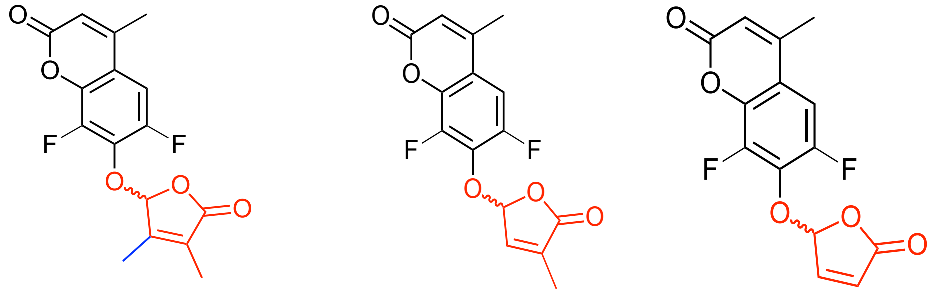

(b)

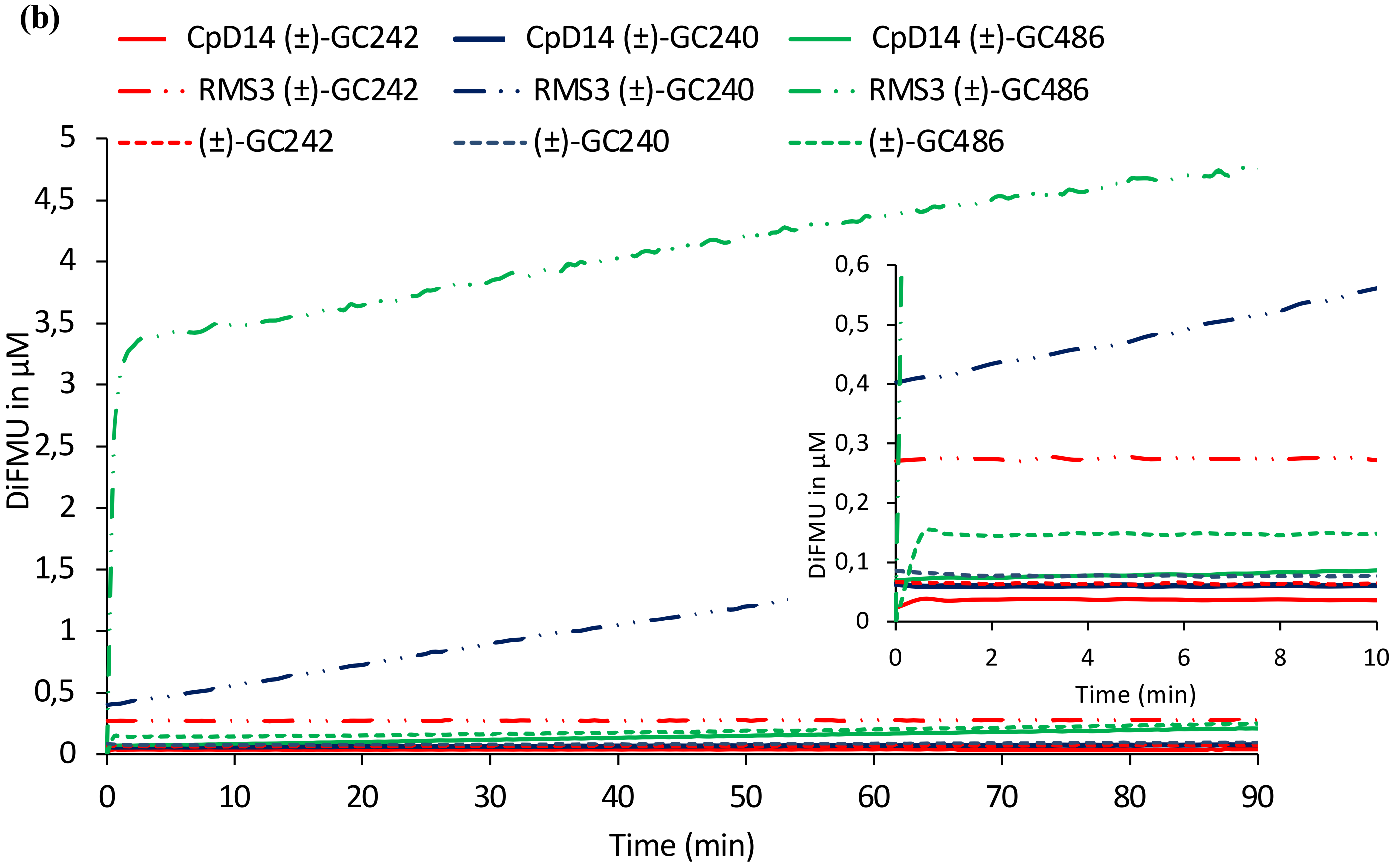

Figure S6. (a) Structures of SL profluorescent probes. (**a**) In black profluorescent part, in red methylbutenolide and desmethylbutenolide D-ring, in blue extra methyl group according to the natural methylbutenolide D-ring found in (±)-GC240. (**b) Enzymatic kinetics for CpD14 (1 µM) and RMS3 (0.33 µM).** Proteins were incubated with the profluorescent probes (±)-GC242, (±)-GC240 or (±)-GC486 at 20 µM. Progress curves during probes hydrolysis, monitored (λ_{em} 460 nm) at 25°C. These plots represent one of the three replicates, and the experiments were repeated twice.

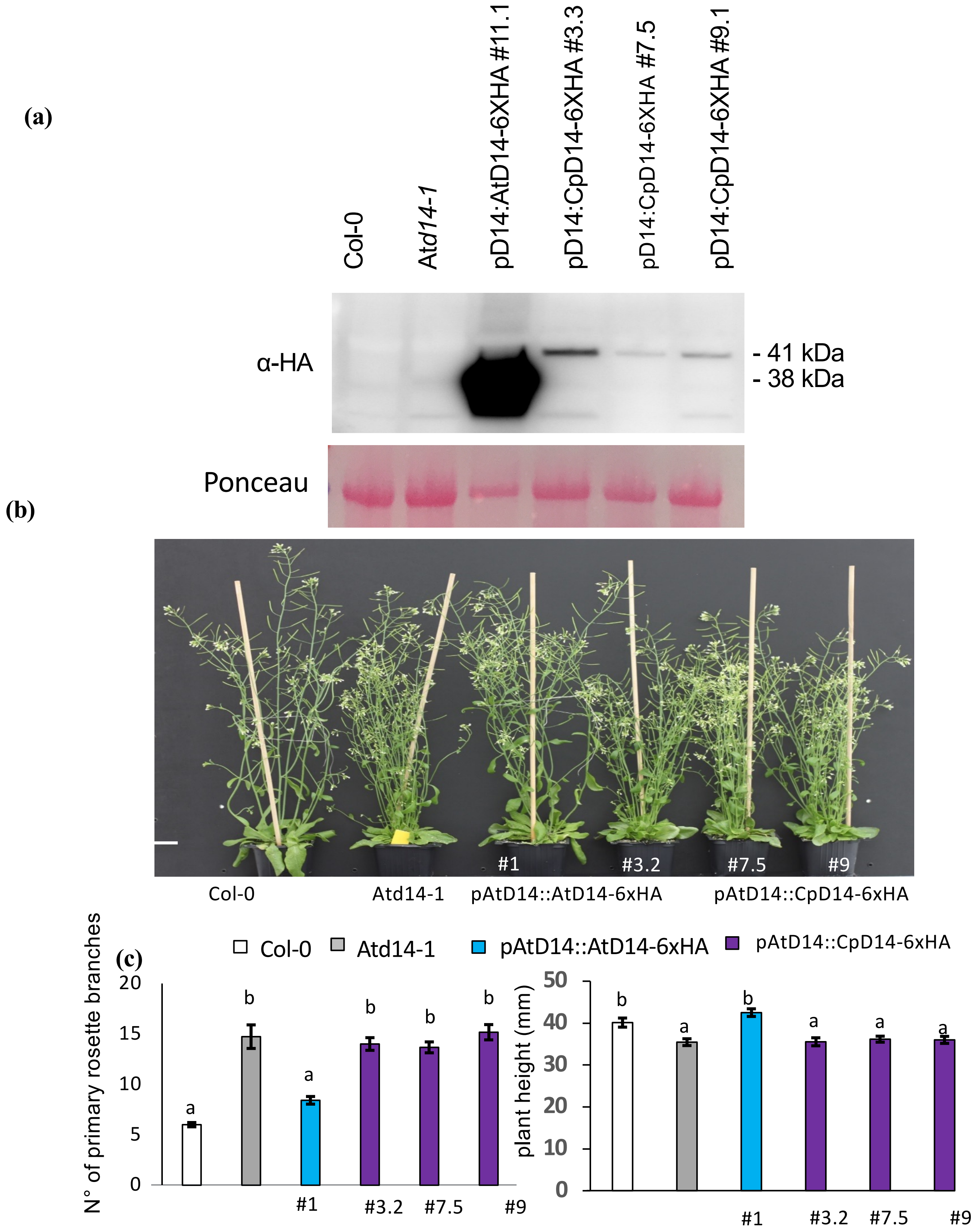

Figure S7. Complementation assay in *Arabidopsis thaliana atd14-1* mutant line. (a) Immunoblot quantification of AtD14-6xHA and CpD14-6xHA using α-HA antibody in *A. thaliana* Col-0 (wt) and *atd14-1* plants, expressing either AtD14 or the CpD14 protein under the control of the *AtD14* promoter. Protein extracts from 40-d-old leaves were separated by 10% SDS-PAGE and identified as a 38 kDa for AtD14 and 41 kDa band for CpD14. Shoot-branching (**b, c**) and plant height (**b, c**) phenotypes of the Arabidopsis *Atd14-1* mutant transformed with a chimeric construct consisting either of the AtD14 coding sequence fused to a 6xHA tag (pAtD14::AtD14-6xHA) or the CpD14 coding sequence fused to the same tag (pAtD14::CpD14-6xHA); both were expressed under the control of the native Arabidopsis *D14* promoter. T3 segregating seeds were grown for 40 days. Data are means ± SE of 12 plants. Letters indicate different statistical groups (ANOVA, post-hoc Tukey test) for $p<0.05$. Further methodological details are provided in Methods. Scale bar = 4 cm. The experiments were repeated twice.

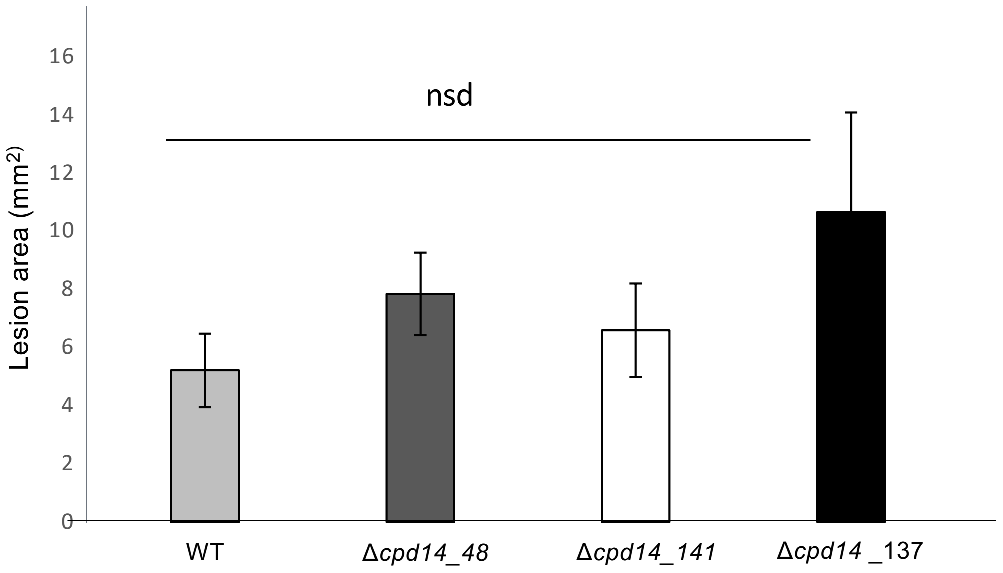

Figure S8. *Cryphonectria parasitica* virulence assay on chestnut cuttings. Three Δ*cpd14* knock-out mutants (Δ*cpd14_48*; Δ*cpD14_141*; Δ*cpD14_137*) were compared to the WT strain *Ku80* to test the ability to induce canker on chestnut cuttings. Lesion area was measured for each isolate. The histogram represents average of lesion area (y axes is shown in Log10 scale). Data are means ± SE of 9 chestnut cuttings. Normal distribution was not detected for the samples; thus, data were treated with the Kruskal-Wallis test observing no statistically significant differences in the distribution of growth areas.
