# Supplementary material for: A structural homologue of the plant receptor D14 mediates responses to strigolactones in the fungal phytopathogen Cryphonectria parasitica
Source: New Phytol. 2022 Feb 26;234(3):1003–17. doi: 10.1111/nph.18013 (PMC9306968; doi:10.1111/nph.18013)
Supplement: Supplementary file 2 — Table S1 List of primers. Please note: Wiley Blackwell are not responsible for the content or functionality of any Supporting Information supplied by the authors. Any queries (other than missing material) should be directed to the New Phytologist Central Office. [file NPH-234-1003-s001.pdf]

Table S1: List of primers

primer name	sequence
KOFor	TGAGCGCAGATGGTAGTGAA
KORev	GCTTTGAGAGGCCGTTTCTT
GibFor	CCAATCCTACCATGAGCAACTGGTTCCCGGTCGGC3
GibRev	CCAGCGATGACAAGCAACTGATATTGAAGGAGCATTTTTTGG
Hyg100For	GACGCCCCAGCACTCGT
CpGapdhFor	GCCACCTACGAGCAGATCAAG
CpGapdhRev	TGGGGTTGCCGTTCATGT
CpTubulinFor	CGCAACGGTCGCTACCTG
CpTubulinRev	TGCGCATCTGGTCCTCG
CpD14LFor	GCCGGTGGAGGTCCCCATG
CpD14LRev	GTACCCAGCAACTACACAACGTTTG
309Rev	GTACCCAGCAACTACACAACGTTTG
CpD14L_attb1_HR	ggggacaagtttgtacaaaaaagcaggctccctggaagtgctgtttcagggcccgATGGCGACAAGTAACACAAC-3‘
CpD14L_attb2	ggggaccactttgtacaagaaagctgggtctcaTCATGCCTTGGACTCAGTC
CpD14LqrtFor	ATGGCGACAAGTAACACAACAA
CpD14LqrtRev	ACAGCGCTCATGGTAGGATTG
CpPhyde-For	CCCGACCAACTACCAGATCTTC
CpPhyde-Rev	TACACTCAGCTCATAATGCCGG
CpPhysi-For	GTGCAGACTTACATCACCTCCA
CpPhysi-Rev	CCAGAATCAGACCCAAGTACGT
Cpoco-For	CCTCACCAAACCCTACCAGTAC
CpocoRev	CGTCGTCTTCCTTCTCACTACC
CpAlde-For	GTTCGACACCTTTACGCACTTC
CpAlde-Rev	GAGGACGAACTTGAGCCAGTAG
CpCarK-For	GTGTATCACGGTTCGGAGTCTT
CpCarK-Rev	AGGCAAGAAGATGAACCGATGT
